# Genomic Characterization of the Genus *Nairovirus* (Family *Bunyaviridae*)

**DOI:** 10.3390/v8060164

**Published:** 2016-06-10

**Authors:** Jens H. Kuhn, Michael R. Wiley, Sergio E. Rodriguez, Yīmíng Bào, Karla Prieto, Amelia P. A. Travassos da Rosa, Hilda Guzman, Nazir Savji, Jason T. Ladner, Robert B. Tesh, Jiro Wada, Peter B. Jahrling, Dennis A. Bente, Gustavo Palacios

**Affiliations:** 1 Integrated Research Facility at Fort Detrick, Division of Clinical Research, National Institute of Allergy and Infectious Diseases, National Institutes of Health, Frederick, MD 21702, USA; kuhnjens@mail.nih.gov (J.H.K.); wadaj@mail.nih.gov (J.W.); jahrlingp@niaid.nih.gov (P.B.J.); 2 Center for Genome Sciences, United States Army Medical Research Institute of Infectious Diseases, Fort Detrick, Frederick, MD 21702, USA; michael.r.wiley19.ctr@mail.mil (M.R.W.); karla.prieto.ctr@mail.mil (K.P.); jason.t.ladner.ctr@mail.mil (J.T.L.); 3 Galveston National Laboratory, Institute for Human Infection and Immunity, Department of Microbiology & Immunology, University of Texas Medical Branch, Galveston, TX 77555, USA; seerodri@utmb.edu (S.E.R.); aptravas@utmb.edu (A.P.A.T.d.R.); hguzman@utmb.edu (H.G.); rtesh@utmb.edu (R.B.T.); dabente@utmb.edu (D.A.B.); 4 Information Engineering Branch, National Center for Biotechnology Information, National Library of Medicine, National Institutes of Health, Bethesda, MD 20892, USA; bao@ncbi.nlm.nih.gov (Y.B.); 5 School of Medicine, New York University, New York, NY 10016, USA; nazir.savji@gmail.com (N.S.)

**Keywords:** *Bunyaviridae*, bunyavirus, nairovirus, Dera Ghazi Khan virus, Erve virus, Ganjam virus, Hughes virus, Qalyub virus, Sakhalin virus, Tunis virus, virus classification, virus taxonomy

## Abstract

*Nairovirus*, one of five bunyaviral genera, includes seven species. Genomic sequence information is limited for members of the *Dera Ghazi Khan*, *Hughes*, *Qalyub*, *Sakhalin*, and *Thiafora nairovirus* species. We used next-generation sequencing and historical virus-culture samples to determine 14 complete and nine coding-complete nairoviral genome sequences to further characterize these species. Previously unsequenced viruses include Abu Mina, Clo Mor, Great Saltee, Hughes, Raza, Sakhalin, Soldado, and Tillamook viruses. In addition, we present genomic sequence information on additional isolates of previously sequenced Avalon, Dugbe, Sapphire II, and Zirqa viruses. Finally, we identify Tunis virus, previously thought to be a phlebovirus, as an isolate of Abu Hammad virus. Phylogenetic analyses indicate the need for reassignment of Sapphire II virus to *Dera Ghazi Khan nairovirus* and reassignment of Hazara, Tofla, and Nairobi sheep disease viruses to novel species. We also propose new species for the Kasokero group (Kasokero, Leopards Hill, Yogue viruses), the Ketarah group (Gossas, Issyk-kul, Keterah/soft tick viruses) and the Burana group (Wēnzhōu tick virus, Huángpí tick virus 1, Tǎchéng tick virus 1). Our analyses emphasize the sister relationship of nairoviruses and arenaviruses, and indicate that several nairo-like viruses (Shāyáng spider virus 1, Xīnzhōu spider virus, Sānxiá water strider virus 1, South Bay virus, Wǔhàn millipede virus 2) require establishment of novel genera in a larger nairovirus-arenavirus supergroup.

## 1. Introduction

With over 530 members, *Bunyaviridae* is one of the largest virus families [1]. Bunyaviruses are characterized by single-stranded RNA genomes that typically consist of separate small (S), medium (M), and large (L) segments, all of which have complementary 3′ and 5′ termini. Most bunyavirus genomes are of negative polarity, but some viruses use ambisense strategies to express their proteins [1,2]. The S, M, and L segments encode the structural nucleoprotein (NP), glycoprotein precursor (GPC), and RNA-dependent RNA polymerase (L) proteins, respectively [1]. Nonstructural proteins are encoded by several, but not all bunyaviruses, by either the S or M or by both S and M segments. Bunyavirions enter host cells by engaging cell-surface receptors with their glycoproteins followed by endocytosis and release of genomes. The viruses typically replicate in the cytosol of infected cells and produce progeny virions that bud from cellular membranes derived from the Golgi apparatus via exocytosis [3].

The family *Bunyaviridae* currently includes five recognized genera: *Hantavirus*, *Nairovirus*, *Orthobunyavirus*, *Phlebovirus*, and *Tospovirus* [1]. Family members have been assigned to these genera, and within genera to species, based primarily on serological cross-reactions, characteristic genus-specific genome segment termini sequences, host association (invertebrates, vertebrates or plants), transmission pathways (arthropod-borne *versus* vertebrate excreta-driven) and, until recently, very limited genomic sequence information [1].

The genus *Nairovirus* includes seven species that are accepted by the International Committee on Taxonomy of Viruses (ICTV) [1]. Most of these species have several distinct members, all of which are either maintained in arthropods or transmitted by ticks among bats, birds, eulipotyphla, or rodents. The most important nairovirus with public-health impact is the tick-borne Crimean-Congo hemorrhagic fever virus (CCHFV), which causes a frequently lethal viral hemorrhagic fever in Western Asia, the Balkans, Southern Europe, and most of Africa [3]. The most important nairoviruses of veterinary importance are the tick-borne Nairobi sheep disease and Ganjam viruses (NSDV and GANV, respectively), which are known to cause lethal hemorrhagic gastroenteritis in small ruminants in Africa and India [4].

The typical nairovirus genome is approximately 18.8 kb in length (S: ≈1.7 kb; M: ≈4.9 kb; L: ≈12.2 kb) and characterized by the genus-specific 3′ segment terminus AGAGUUUCU and 5′ segment terminus AGAAACUCU. Classical nairovirions are enveloped spheres (80–120 nm in diameter) spiked with heterodimeric glycoprotein projections consisting of the cleavage products of the glycoprotein precursor (Gn and Gc) [3].

Next-generation sequencing followed by coding-complete or complete genomic sequence assembly (see [5] for sequencing nomenclature) is increasingly used to classify previously uncharacterized phleboviruses [6,7,8,9,10,11,12,13,14] and orthobunyaviruses [15,16,17,18,19,20,21] and to characterize novel bunyavirus clades, such as “goukoviruses,” “herbeviruses,” “phasmaviruses,” and the Ferak and Jonchet virus groups [22,23,24]. Several unclassified bunyaviruses and viruses assigned to bunyaviral genera other than *Nairovirus* have been identified as bona fide nairoviruses [25,26,27,28,29,30,31,32,33,34]. At least one classified nairovirus was identified as an actual phlebovirus [14]. Novel nairoviruses have been discovered in bats [25,27,29,35,36], and in arachnids, millipedes, and water striders [37,38,39,40]. Even more interestingly, at least two nairo-like viruses with only bisegmented genomes have been reported [37,41]. Shortly before this manuscript was submitted, Walker *et al.* reported the coding-complete sequences of 11 nairoviruses (Abu Hammad virus (AHV), Avalon virus (AVAV), Bandia virus (BDAV), Dera Ghazi Khan virus (DGKV), Erve virus (ERVEV), Farallon virus (FARV), GANV, Punta Salinas virus (PSV), Qalyub virus (QYBV), Taggert virus (TAGV), and Zirqa virus (ZIRV)) [42]. An overview of all viruses currently thought to be nairoviruses or nairo like-viruses, and their relationships based on data prior to this study are provided in Table S1.

As is evident from the table, genomic sequence information for nairoviruses is still limited. Here we report either the coding-complete or complete genomic sequences of 23 nairoviruses (Table 1). Ten of these sequences have also been determined by Walker *et al.* [42]. Four of the 23 sequences are for novel strains of previously sequenced nairoviruses. Nine of the 23 sequences are new from previously unsequenced viruses. We extended 14 sequences to include all of the 3′ and 5′ genome segment termini. Our subsequent phylogenetic analyses indicate a number of changes in the organization of nairoviruses. At least five new nairovirus species ought to be established. GANV should be considered an isolate of NSDV, and soft tick bunyavirus should be considered an isolate of Keterah virus (KRTV). Tunis virus (TUNV), which was serologically identified as a phlebovirus, is an isolate of AHV in the *Dera Ghazi Khan nairovirus* species. At least seven nairo-like viruses should be classified into novel genera, and these genera and all nairoviruses are more closely related to arenaviruses than to other bunyaviruses.

## 2. Materials and Methods

### 2.1. Viruses

The viruses used in this study were obtained from the World Reference Center for Emerging Viruses and Arboviruses at the University of Texas Medical Branch, Galveston, TX, USA. All of these viruses have been described before. Table 1 provides specifics about the viruses and GenBank accession numbers for all newly sequenced and re-sequenced viruses.

### 2.2. Genome Sequencing

Viral stocks were obtained in TRIzol LS (Invitrogen, Carlsbad, CA, USA), and RNAs were extracted using the Direct-zol™ RNA MiniPrep kit (Zymo, Irvine, CA, USA). RNAs were converted to cDNAs and amplified using sequence-independent single primer amplification as described previously [65] with some modifications to resolve the 5′ and 3′ ends. An oligonucleotide containing three ribonucleotides (rGTP) at the 3′ end (GCCGGAGCTCTGCAGATATCGGCCATTATGGCCrGrGrG) and the FR40RV-T primer [65] were added during first-strand cDNA synthesis. The reverse transcriptase was changed to Maxima H Minus reverse transcriptase (Thermo Fisher Scientific, Waltham, MA, USA), which has terminal transferase activity that adds the rGTP-containing oligonucleotide to the 5′ end during cDNA synthesis. cDNA was sheared to ≈400 bp in length and used as starting material for creation of Illumina TRUseq DNA libraries. Sequencing was performed on either an Illumina MiSeq or NextSeq desktop sequencer using 300-cycle kits (2 × 150). Open-source Cutadapt [66] and Prinseq-lite [67] were used to trim primers and remove poor quality reads, respectively. Reads were assembled into contigs using open-source Ray Meta [68]. Annotation was determined using basic local alignment search tool (BLAST) in combination with custom scripts. Contigs related to nairovirus sequences were used as references.

### 2.3. Phylogenetic Analysis

A set of nairovirus sequences (252 for the N gene of the S segment, 111 for the M segment, and 93 for the L segment) comprising the majority of the nucleotide (nt) sequences from GenBank available on 1 March 2016, were aligned using the CLUSTAL algorithm. Because the nairovirus sequences of all analyzed nairoviruses were so different that the alignment reached substitution saturation (no detection of signal), alignments were instead implemented at the amino acid (aa) level (using MEGA Version 5 [69]). Non-coding regions of S segments therefore had to be excluded. Additional manual editing was performed to ensure the highest possible quality of alignments. Neighbor-joining (NJ) analysis at the aa level was performed due to the observed high variability of the underlying nt sequences. The statistical significance of tree topology was evaluated by bootstrap re-sampling of the sequences 1000 times. Phylogenetic analyses were performed using MEGA Version 5.

### 2.4. Detection of Reassortant Events

Systematic screening for the presence of recombination patterns was pursued by using the nt alignments and the Recombination Detection Program (RDP [70]), Bootscan [71], maximum chi-square (MaxChi) [72], Chimaera [73], Likelihood Analysis of Recombination in DNA (LARD) [74], and Phylip Plot [75].

### 2.5. Sequence Analysis

Geneious 4.8.3 (Biomatters Inc., Newark, NJ, USA) was used for sequence assembly and analysis. Topology, sizes, and targeting predictions were generated by employing SignalP 4.1, NetOGlyc 4.0, NetNGlyc 1.0, Prop 1.0, tied mixture hidden Markov model (TMHMM) 2.0 [76], SnapGene Viewer 2.82 [77], the web-based version of TopPred2 [78], and integrated predictions in Geneious [79,80,81,82,83].

## 3. Results

### 3.1. Genomic Characterization and Phylogenetic Analysis

**Figure 1:**
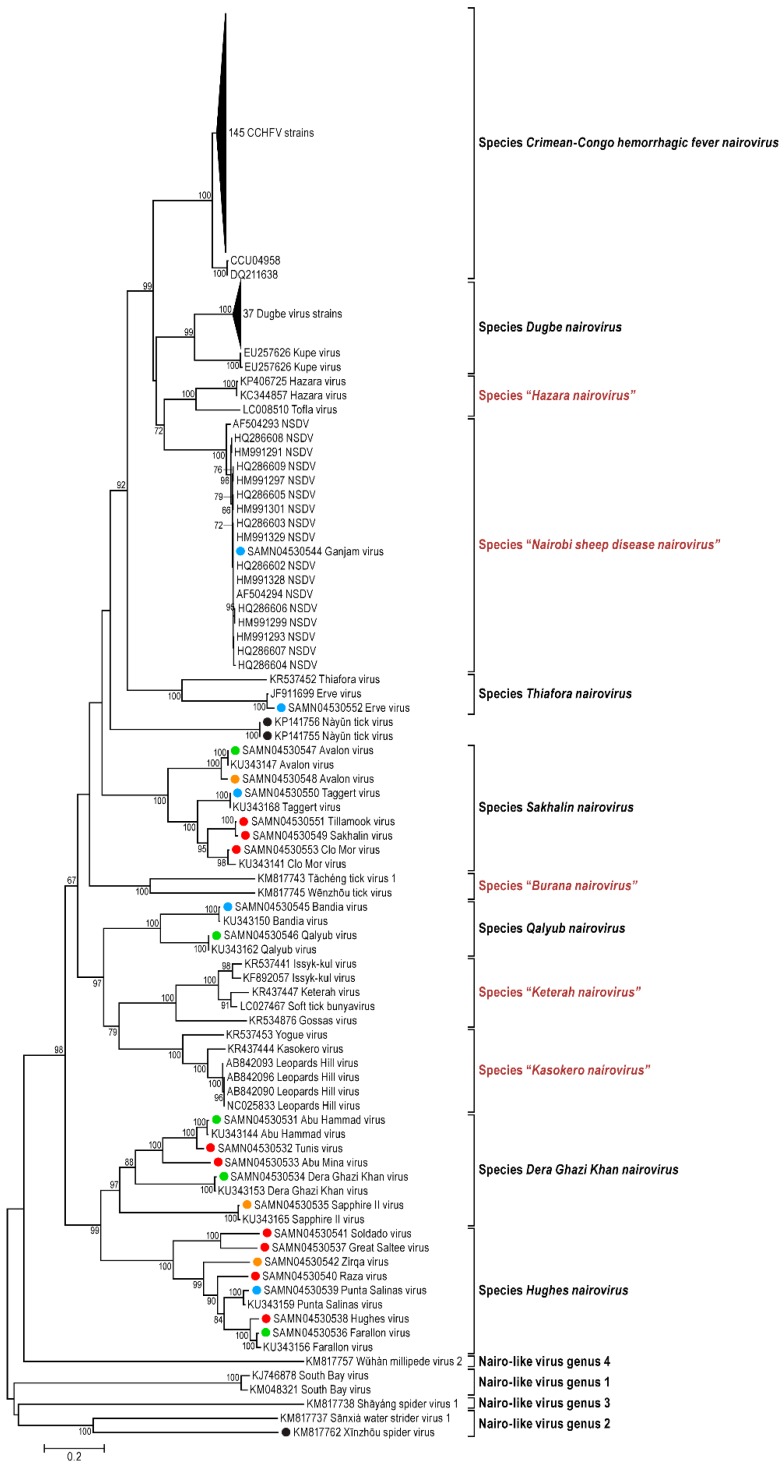
Phylogenetic analysis of nairovirus and nairo-like virus S segment N gene sequences, including newly determined virus sequences (red dots), newly determined virus isolate sequences (orange dots), re-sequenced genomes (blue dots), and re-sequenced genomes with genomic termini determined for the first time (green dots). Sequences marked with black dots correspond to partial sequences. Nairovirus sequences comprise all partial or complete sequences from GenBank available on 1 March 2016. Proposed new taxa are highlighted in red and placed in quotation marks.

**Figure 2:**
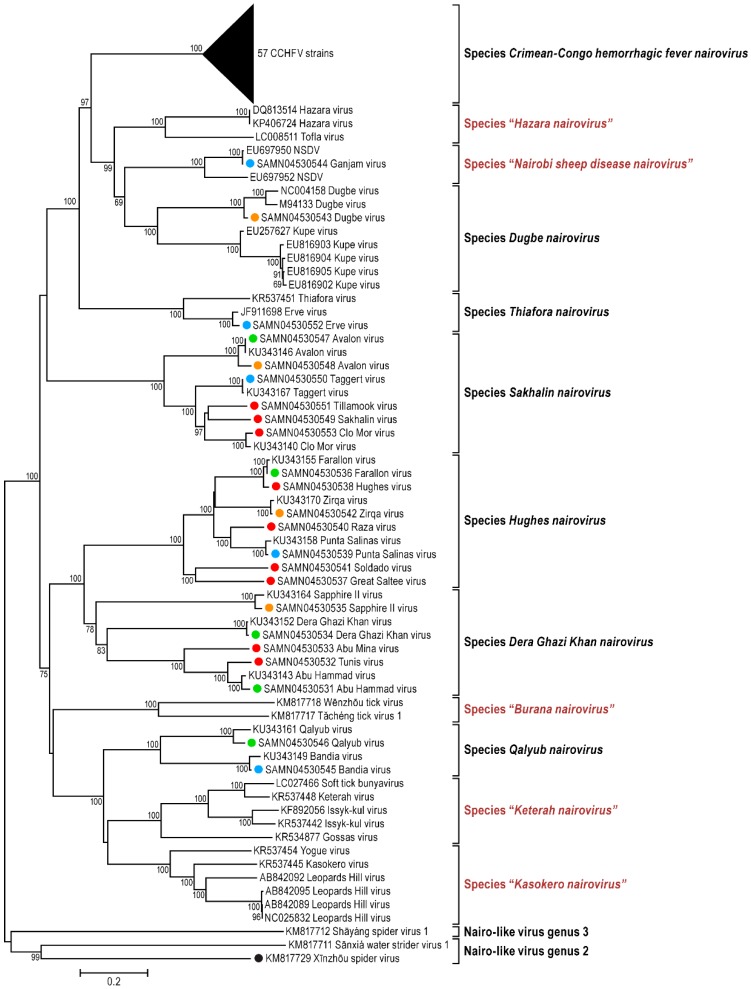
Phylogenetic analysis of nairovirus and nairo-like virus M segment sequences. Analysis was performed as outlined for Figure 1.

**Figure 3:**
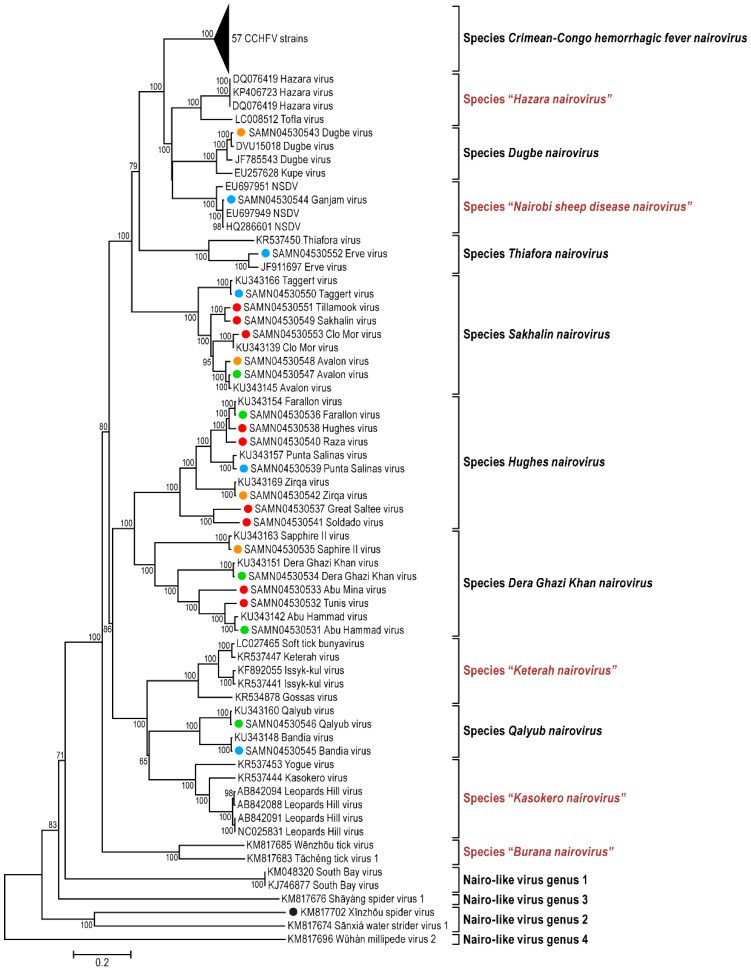
Phylogenetic analysis of nairovirus and nairo-like virus L segment sequences. Analysis was performed as outlined for Figure 1.

Consistent with the genomic organization characteristic for already sequenced nairoviral genomes, each of the 23 viral genomes sequenced during this study is comprised of three RNA segments including (a) a small (S) segment encoding the NP and, in an ambisense orientation, a non-structural protein (NSs); (b) a medium (M) segment encoding a GPC; and (c) a large (L) segment encoding an RNA-dependent RNA polymerase. Fourteen nairovirus genomes were completely characterized. The 3′ terminal sequences were obtained for 57 segments, and the 5′ terminal sequences were obtained for 51 segments (Table 1). For most of the viral genomes sequenced in this study, the nine most terminal nucleotides of each segment were identical to those previously reported for nairoviruses (3′ segment terminus AGAGUUUCU and 5′ segment terminus AGAAACUCU) [1,3]. However, the Abu Hammad virus (AHV), Abu Mina virus (AMV), Dera Ghazi Khan virus (DGKV), Sapphire II virus (SAPV), and Tunis virus (TUNV) genome segments have termini that differ by one nt (AGAGUUUCA and TGAAACUCU). Likewise, the Qalyub virus (QYBV) genomic segments termini differ from the consensus sequences by one nt (AGAGAUUCU and AGAATCUCU).The results of phylogenetic analyses of the newly obtained L, M, and S segment sequences are shown in Figure 1, Figure 2 and Figure 3.

The phylogenetic placement of the newly sequenced viruses is largely consistent with their previous serological classification, including recent amendments [42] (Table S1). However, Hazara virus (HAZV) and Tofla virus (TOFV) clustered with each other but not with CCHFV and, therefore, should not be classified in the species *Crimean-Congo hemorrhagic fever nairovirus*. Likewise, both Kupe virus (KUPEV) and Nairobi sheep disease virus (NSDV) did not cluster with Dugbe virus (DUGV), and, therefore, should be removed from the species *Dugbe nairovirus* and re-assigned to new species (here proposed as “*Hazara nairovirus*” (HAZV, TOFV)) and “*Nairobi sheep disease virus*” (NSDV), respectively). Ganjam virus (GANV) is clearly identified as an isolate of NSDV. Our analysis confirm that Leopards Hill virus (LPHV), Kasokero virus (KAS(O)V), and Yogue virus (YOGV) form a novel nairovirus genogroup (proposed species “*Kasokero nairovirus*”), as do Keterah virus (KRTV) and Issyk-kul virus (ISKV) (proposed species “*Keterah virus*”) [29,42]. The recently described soft tick bunyavirus [38] is identified as an isolate of KRTV. Genetic characterization of TUNV clearly demonstrates that this virus is a nairovirus and not a phlebovirus as previously described by serological analysis [63]. The TUNV genome represents an isolate of AHV, indicating necessary classification into the species *Dera Ghazi Khan nairovirus*. Another new species, proposed to be named “*Burana nairovirus*” should be established for Wēnzhōu tick virus, Huángpí tick virus 1, and Tǎchéng tick virus 1. Finally, the phylogenetic trees demonstrate that several nairo-like viruses with three (Shāyáng spider virus 1, Xīnzhōu spider virus (XSV), Sānxiá water strider virus 1 (SWSV-1), South Bay virus (SBV)) or two genomic segments (SBV, Wǔhàn millipede virus 2) should not be classified in the genus *Nairovirus*.

Although genomic segment reassortment has been found very frequently among CCHFV strains and lineages [84,85,86,87,88], we were unable to detect any instance of reassortment among the other nairoviruses using RDP, Bootscan, MaxChi, LARD and Phylip Plot. Phylogenetic incongruence was only detected in the case of HAZV: whereas the HAZV M and N open reading frames (ORFs) cluster together with those of NSDV/KUPEV, the HAZV L ORF does not. However, given the genetic distance between these sequences, whether this distance is the result of reassortment or saturation of the phylogenetic signal is not clear.

### 3.2. Open Reading Frames

#### 3.2.1. Small (S) Segment—Nucleocapsid Protein

Nairoviral NPs may recognize specific nairoviral RNA sequences, bind non-specifically to single-stranded RNA, and form the ribonucleoprotein (RNP) complex [89,90,91,92,93,94]. The structure of NP has been determined for CCHFV, ERVEV, HAZV, and KUPEV [89,92,93,94]. All nairovirus NPs assume a racket-shaped structure with distinct “head” and “stalk” domains that are typical for bunyaviruses and unique among other negative-sense single-stranded RNA viruses.

In the case of CCHFV NP, two positively charged regions are responsible for RNA binding [92]. One region forms a large positively charged crevice (residues K339, K343, K346, R384, K411, H453, and Q457), of which two residues contribute to a conserved nairovirus motif (EH_453_(L/M), (L/F)HQ_457_). The other region is delineated by residues R134, R140, and Q468. The CCHFV NP stalk region also contains a positively charged region consisting of residues R195, H197, K222, R225, R282, and R286. Only three of the positively charged residues (R134, K222 and K343) are absolutely conserved among all nairovirus NPs, although most of the substitutions observed maintain the overall hydrophobicity profile.

CCHFV NP interacts with the antiviral defense factor MxA [95] and the apoptosis mediator caspase-3 [96]. Thus, we expected some degree of conservation of the NP areas mediating those functions. Using a CCHFV minireplicon system [89], three separate NP residues (K132, Q300, and K411) were identified to be essential for replicon activity, and mutation of another two residues (K90 and H456) resulted in significantly reduced NP functionality. However, only H456 and Q300 are completely conserved among nairovirus NPs.

**Figure 4:**
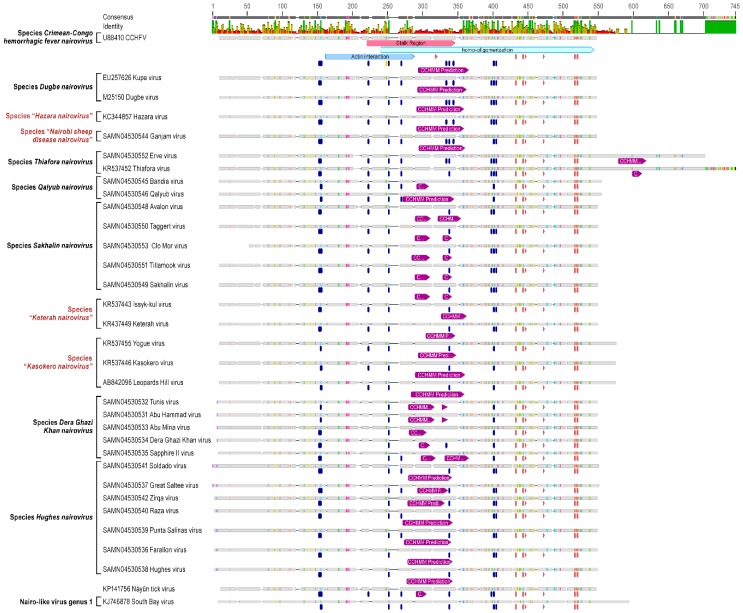
Nairoviral nucleoproteins (NPs) similarity plot comparing typical features of nairoviral NPs. The NP sequence of Crimean-Congo hemorrhagic fever virus (CCHFV) is taken as the reference sequence. Ovals in orange and blue highlight two regions responsible for RNA binding.

Because protein structure is evolutionarily conserved to a higher degree compared to the primary aa sequence, we used homology modeling principles and techniques to identify conserved structures among nairovirus proteins. We used the Phyre2 (Protein homology/analogy recognition engine) server to model the structure of the proteins and to align remotely related sequences based on hidden Markov models (HMMs) (Figure 4).

In addition, upon identification of a conserved nairovirus protein structure, we performed mutational sensitivity analysis using the Disease-Susceptibility-based SAV Phenotype Prediction (SuSPect) tool [97], to predict whether missense mutations in a nairovirus protein are likely to functionally affect structure.

**Figure 5:**
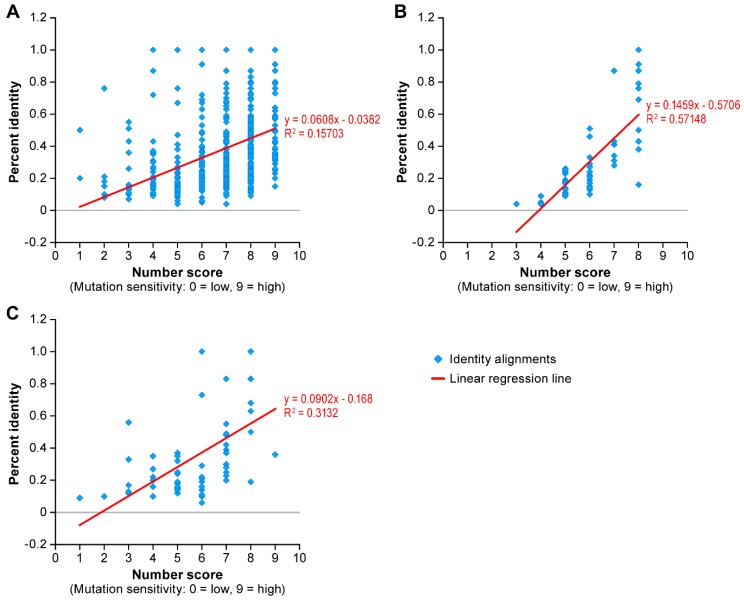
Mutational Sensitivity Analysis. The mutational sensitivity number score for each nairovirus NP amino-acid position (SuSPect) was plotted against the percent identity for that position in the nairovirus NP protein sequence alignment. (**A**) Full-length nairovirus NP; (**B**) myosin-4 motor protein-like NP domain; (**C**) ovarian tumor (OTU) domain of nairovirus L.

Finally, the mutational sensitivity score was plotted for each position against the average percent identity for that position in the nairovirus protein alignment. We assumed that if a mutation would have an influence on the structure of a conserved domain, the frequency of that mutation would be diminished. In summary, we expected that the positions with a higher score in the mutational sensitivity analysis should directly correlate with positions of higher conservation. The validity of this approach was tested initially with nairovirus NP sequences (Figure 5A,B). As expected, all nairovirus NPs were found to be structurally homologous to CCHFV NP, and the positional analysis revealed a direct correlation between positions of high mutational sensitivity with those of high identity (Figure 5A).

Interestingly, we identified two other nairovirus NP domains that were structurally similar to other known, non-viral structures. The first domain, at approximate position 150–200 of the nairovirus alignment, is similar to the globular tail of myosin-4 motor protein (type V myosin; confidence 60%; Protein Data Bank (PDB) ID: 3mmi). Myosin-4 motor protein is a monomeric myosin with motility uniquely adapted to transport mRNA [98]. Interestingly, SuSPect analysis suggested that the domain was sensitive to aa changes (Figure 5B). The second domain, located at the C-terminal NP domain (approximately at positions 460–481), is similar to the structures of the cholesterol-binding toxins intermedilysin (confidence 35%; PDB: 1s3r), perfringolysin (PDB: 1pfo) and pneumolysin (PDB: 4qqq), but SuSPect analysis did not show mutational sensitivity at these positions (data not shown).

#### 3.2.2. Medium (M) Segment—Glycoprotein Precursor

ORF analysis of 36 sequenced nairovirus M segments generally yielded single unit polyprotein-encoding ORFs in each case (two units in the case of “*Burana nairovirus*” M segments). The predicted masses of the encoded unmodified polyproteins, the GPCs, ranged from 143 kDa (Thiafora virus (TFAV)) to 187 kDa (CCHFV). Each nairovirus polyprotein was approximated, via software modeling, to contain a signal peptide at the N-terminus. Experimental evidence garnered from CCHFV GPC processing [99,100,101] was used for functional element assignment in predicted nairovirus GPCs. Cleavage motifs for signal peptidases, furin (RSKR), subtilase SKI-I/S1P-like (RRLL and RKLL), and an unknown convertase that cleaves at the aa sequence RKPL, are highly conserved across CCHFV isolates and are critical post-translational motifs in the viral lifecycle [99,100,101,102]. Interestingly, the RSKR motif appears to be unique to CCHFV. The RKLL motif for SKI-I/S1P protease is conserved among nairovirus GPCs and is found in most members of all established and putative nairoviruses except in AVAV, DUGV, ERVEV, KAS(O)V, KUPEV, NSDV/GANV, and YOGV. The RKLL motif is not confined to any specific region/domain and occurs throughout the nairovirus GPCs—in some instances more than once (e.g., DUGV and members of *Qalyub nairovirus* and “*Keterah nairovirus*”). The RRLL motif is the second most prevalent cleavage site with the exception of viruses belonging to “*Keterah nairovirus*” and *Qalyub nairovirus*, which do not contain the motif. The RKPL and RRLL motifs are also conserved across nairoviruses of many species.

**Figure 6:**
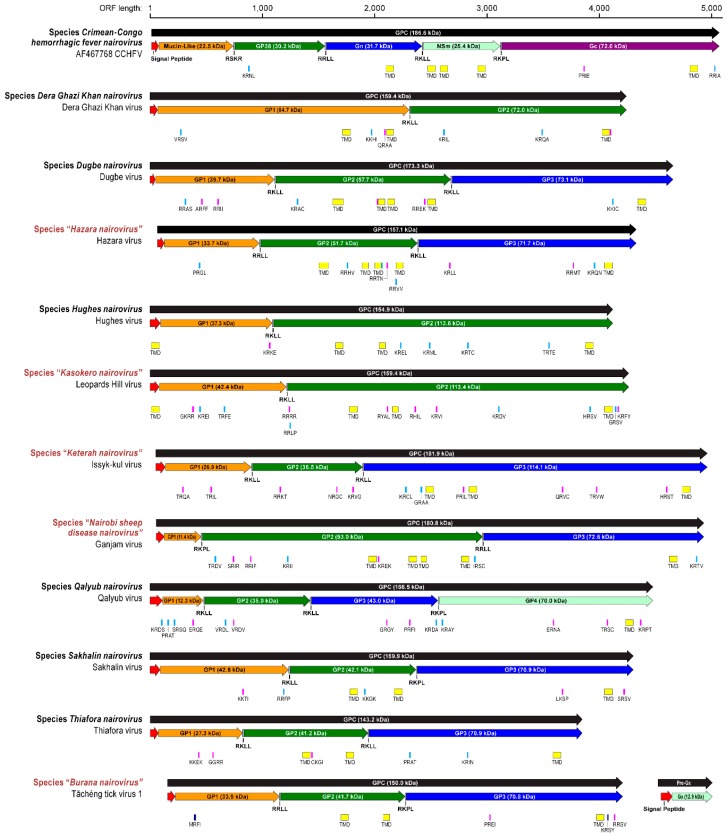
Nairovirus M segment-encoded polyprotein features and annotations. Type virus glycoprotein precursors (GPC) are represented for each species as black arrows. Putative glycoproteins (GP) are designated based on experimentally proven cleavages of CCHFV GPC (indicated in grey boxes separating glycoproteins). GPs are designated as colored arrows and numerically annotated beginning from the N-termini. The predicted molecular weights (kDa) of GPCs and putative GPs are annotated within colored arrows. Molecular weights were predicted without glycosylation. No designations of structural *versus* non-structural (Gn/Gc *vs.* NSm) proteins are listed due to lack of available experimental evidence across the genus. Signal peptides were predicted using posterior probability thresholds of 1.0 and 0.1 (SignalP 4.1) and are annotated with red arrows. Proprotein cleavage predictions (Prop 1.0) were analyzed for general convertase and furin predictions. Proprotein cleavage prediction thresholds of 0.3–0.49 are annotated with pink boxes, and thresholds of 0.5 or higher are annotated in sky blue. For both predictions, four-letter amino-acid sequences are provided below each colored box. Transmembrane domains (TMD) are annotated with yellow boxes and predicted using TMHMM 2.0. Two members of the species “*Burana nairovirus*” were predicted to have two open reading frames on the M-segment encoding a separate stand-alone glycoprotein (Pre-Gx) next to the polyprotein (GPC).

Using the four cleavage motifs (RSKR, RKPL, RRLL, RKLL), the nairovirus GPs stemming from GPC processing were predicted and annotated numerically, starting at the N-termini and depicted as colored arrows (Figure 6). We predicted the synthesis of two to five GPs depending on the examined nairovirus (Table S2 and Figure 6). To further explore GPC processing phenotypes, proprotein convertase prediction software was used to identify additional cleavage patterns. The most prevalent motifs predicted with a high degree of probability to mediate post-translation cleavage along the GPC were R-X-X-L, G-X-X-R, Q-X-X-C, and R-X-X-K, (data not shown and Figure 6). Other predicted motifs (with varying degrees of probability) are shown in sky blue and pink boxes within Figure 6.

Other post-translational modifications, such as, *N*- and *O*-linked glycosylations, were predicted for all analyzed nairoviruses using software modeling (NetOGlyc 4.0, NetNGlyc 1.0). Averages of *N*-linked glycosylation sites between viruses of different species ranged from seven to twenty sites along polyproteins. The most *N*-linked glycosylations were found for members of the “*Keterah nairovirus*” and *Sakhalin nairovirus* species, whereas the members of the *Dugbe* and *Hazara nairovirus* species had the fewest. The extent of nairovirus glycoprotein *O*-linked glycosylation ranged from four to over 130 sites (Table S2). The fewest *O*-glycans were predicted for members of the *Hughes nairovirus* and *Dera Ghazi Khan nairovirus* species, ranging from four to 17 sites. Intermediate *O*-linked glycosylation was predicted for *Hazara*, *Dugbe*, *Qalyub*, *Sakhalin*, and *Thiafora nairovirus* species members, with averages that ranged from 29 to 48 sites. The highest number of *O*-glycans (100 and 135 sites, respectively), were predicted for viruses of the *Crimean-Congo hemorrhagic fever nairovirus* and “*Keterah nairovirus*” species. Relatively few sites were predicted towards the C-termini of nairovirus GPC, with the exception of the “*Keterah nairovirus*” species members, which were predicted to contain a small cluster of *O*-linked sites between residues 994 and 1033.

Heavily *O*-glycosylated GPCs were typically characterized by site clustering towards the N-termini. O-glycosylated members of the *Dugbe*, “*Keterah*,” and *Qalyub nairovirus* species contain RKPL and RKLL proteolytic motifs in these areas. If used by proteases, these motifs could mediate the production of separate, stand-alone peptides that are *O*-glycosylated. Such peptides have been identified in the cases of CCHFV and DUGV as mucin-like domains (MLDs) [103]. Additionally, viruses of the “*Leopards Hill nairovirus*” species encode GPC with shorter regions of *O*-linked glycosylation clustered towards the N-termini in the vicinity of predicted proteolytic cleavage sites. Of nairovirus genus members containing regions of O-glycosylation/MLDs, unmodified averaged masses ranged from eight to 37 kDa. Notably, of the GPC of seven viruses belonging to the *Hughes nairovirus* species, none were predicted to be *O*-glycosylated.

We also analyzed the nairovirus GPC for the occurrence, location, and topology of transmembrane regions using software modeling (TopPred2). The number of TMDs varied between one and five domains. Interestingly, viruses of the *Qalyub nairovirus* species had the fewest transmembrane regions in their glycoprotein precursors (e.g., QYBV has only one such region). By comparison, CCHFV and DUGV GPCs have five TMDs. All nairovirus GPCs were predicted to have at least a single conserved C-terminal transmembrane region approximately 40–60 residues prior to the C-termini of the GPC (Figure 6 and Table S2).

#### 3.2.3. Large (L) Segment—RNA-Dependent RNA Polymerase

**Figure 7:**
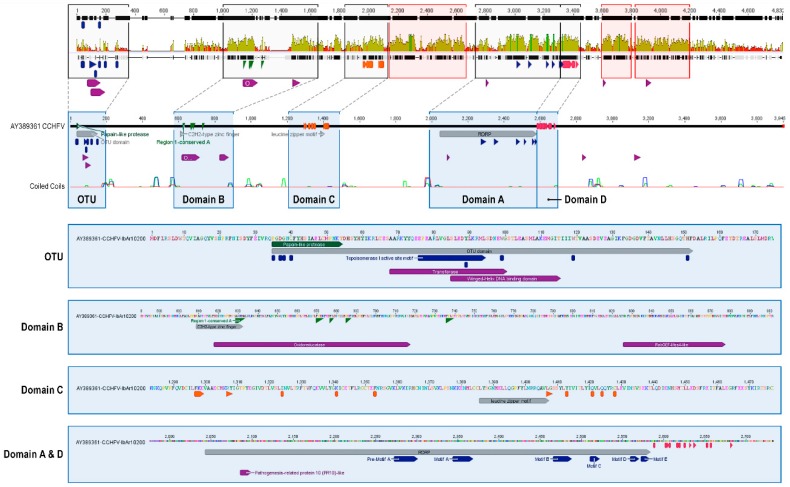
Cartoon showing conserved regions in nairovirus RNA-dependent RNA polymerases (Ls) using CCHFV as a reference. OTU, ovarian tumor family-like domain.

Nairovirus RNA-dependent RNA polymerases (Ls) are substantially larger than other bunyavirus L homologs. All nairovirus L sequences maintain the characteristic RNA-dependent RNA polymerase core motifs described by Poch [104] and Muller *et al.* [105] comprising residues 2361–2669 of the L gene alignment (domain A), and therefore include the polymerase module pre-A motif through motif E (Figure 7).

**Figure 8:**
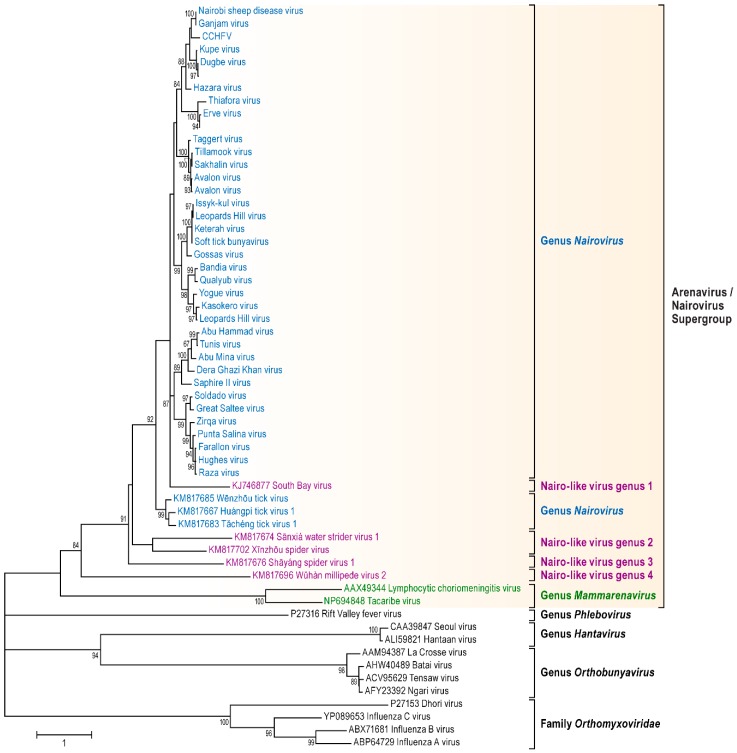
Phylogenetic analysis of the RNA-dependent-RNA polymerase core domain. A set of 58 core domains, comprising motifs A through E for representative viruses were analyzed by maximum likelihood method at the amino acid level using PHYML.

Moreover, inter-motif regions are moderately conserved, suggesting structural constrains on their three-dimensional arrangements. The invariant sequences DXX KW and SDD of motifs A and C, respectively, may have metal-binding activities necessary for catalytic functions [106,107] (Figure S1). A phylogenetic analysis of the nairovirus and nairo-like virus core polymerase modules with the corresponding regions of other bunyaviruses (hantaviruses, orthobunyaviruses, phleboviruses), mammarenaviruses, and orthomyxoviruses is shown in Figure 8.

Our analysis demonstrates that the nairovirus and nairo-like virus RNA-dependent-RNA polymerase core domain is more closely related to the arenavirus domain than to any other bunyaviral domain, supporting the existence of an arenavirus-nairovirus supergroup.

CCHFV encodes a deubiquitinase (DUB) of the OTU family, which unlike eukaryotic OTU DUBs, also targets interferon-stimulated gene 15 (ISG15) modifications [108,109]. The catalytic motifs characteristics of OTU-like cysteine proteases are clearly detected in all nairoviruses, but not in any of the nairo-like viruses. The role of the CCHFV OTU-like cysteine protease in the cleavage of host cell proteins, and specifically on the ubiquitin- and ISG15-dependent innate immune response, has been widely investigated [108,109]. Several OTU-characteristic residues (P35, D37, G38, C40, Y89, W99, W119, and H151) are highly conserved among all nairoviruses (Figure S7-2). Interestingly, these residues are not conserved in any of the nairo-like viruses [110]. P35, D37, G38, C40, and H151 are part of OTU’s catalytic site (Figure S2). Y89 is the key aa residue of a conserved site resembling a topoisomerase motif (SXXXY), but its serine residue is not conserved among nairoviruses. Interestingly, the conserved site Y89 is located very close to P77, E78, and R80, which are key residues for the interaction with ubiquitin and ISG15 [111]. Moreover, this area of the nairovirus OTU domain is structurally similar to the catalytic domain of a transferase-like protein (PDB ID: 1k98; methionine synthase protein, confidence 60.8%) and to the DNA/RNA-binding 3-helical bundle fold of “winged helix” DNA-binding domain proteins. Interestingly, the region in the methionine synthase protein identified as structurally similar to the nairovirus OTU domain is the binding domain for vitamin B12. As expected, positional analysis revealed a correlation between positions with high mutational sensitivity and high identity (Figure 5C). The presence of the conserved OTU motif in all nairovirus Ls and the structural similarities between topoisomerases and strand-specific recombinases could indicate a role of the OTU domain in RNA strand manipulation.

The majority of nairoviruses pathogenic to humans (CCHFV, DUGV virus, ERVEV, ISKV, KAS(O)V, and NSDV) or other mammals (NSDV/GANV) contain an identifiable topoisomerase I active site motif, whereas most nairoviruses without that domain are only known to infect arthropods or to establish subclinical infections in vertebrates. The deubiquitinylation and deISGylation activities of CCHFV L have been proposed as a mechanism of virus evasion from the innate immune response via efficient interference with antiviral signaling pathways mediated by nuclear factor (NF)-kB, interferon regulatory factor 3 (IRF3), and type 1 interferon (IFN-α/β) [108,109]. These pathways rely on protein ubiquitinylation for their activation—one outcome is the modification of the pathway factor with ISG15. Thus, researchers posit that the CCHFV OTU domain might be an important virulence determinant, as some differences were observed in the functionality of the domain in very virulent CCHFV and the less virulent DUGV [112]. The observation of a potential structural and phylogenetic difference between the nairovirus OTUs of pathogenic and non-pathogenic nairoviruses is therefore intriguing.

Position-specific iterated (PSI)-BLAST searches had previously demonstrated that the region between the nairovirus OTU-like cysteine protease domain and the core region of the RNA-dependent polymerase (domain A) is conserved [42,113] (Figure 7). A region, including the C_2_H_2_-type zinc finger domain and several aa positions conserved among all RNA-dependent RNA polymerase modules of segmented negative-sense RNA viruses, was named domain B [106,107] (Figure 7 and Figure S3). Interestingly, this area of L is structurally similar to an oxidoreductase (PDB: 1mv8, a GDP-mannose 6-hydrogenase; positions 328–433; confidence 86.5%: identity 64%). Although several aa residues essential for the proper structure and function of this domain are conserved among nairovirus and other bunyavirus Ls, no correlation between mutational sensitivity and conservation was detected (data not shown). Downstream, Phyre2 analysis identified an area with similarity to the mammalian suppressor of yeast Sec 4 (Mss4)-like superfamily of proteins, of the family Rab guanine nucleotide exchange factor (GEF) Mss4 (PDB: 2fu5, confidence 67.4%, identity 44%). Interestingly, this area also contains several aa residues that are highly conserved among all nairoviruses (Figure 7 and Figure S3). However, no correlation with mutational sensitivity was detected (data not shown).

A region immediately upstream of the core polymerase region is highly conserved among arenaviruses and bunyaviruses (domain C) (Figure 7 and Figure S4; residues conserved among families are highlighted in orange in the figure) [114]. In the case of CCHFV, domain C includes a leucine zipper motif, which is found in all nairovirus genomes. Leucine zipper domains are a common three-dimensional structural motif of transcription factors, characterized by a periodic repetition of leucine residues at every seventh position over a distance covering eight helical turns [115]. The polypeptide segments containing these periodic arrays of leucine residues were proposed to exist in an alpha-helical conformation, and the leucine side chains from one alpha helix interdigitate with those from the alpha helix of a second polypeptide, facilitating dimerization. Basic-region leucine zippers (bZIPs) are a class of eukaryotic transcription factors including leucine zipper domains of 60 to 80 residues in length with highly conserved DNA binding basic regions [116]. The nairovirus leucine zipper domain surrounds a highly conserved NRRQ domain in the center of the 7 moderately conserved leucine positions. Of note, this motif is also structurally similar to human immunodeficiency virus 1 Nef (PDB: 2xi1, positions 97–110, confidence 11%, identify 43%), which includes the functional conserved motif XR [117,118]. Interestingly, structural analysis of nairovirus domain C revealed a similarity to RNA-binding an endoribonuclease VapD (PDB: 3ui3, positions 69–132, confidence 45.6%, identity 40%).

Finally, domain D, which has been previously described only for orthobunyaviruses (Figure 7 and Figure S5; residues conserved among family members are highlighted in orange in the figure), could also be identified as a conserved feature of nairovirus Ls, but structural similarities could not be detected.

Three additional, new conserved domains (Figure 7; orange boxes) were found in the nairovirus L alignment (1424–1605; 1700–1898; and 2763–3368). Only the third (and largest) domain (aa 3122–3155) was found to be structurally similar to another domain, namely the homodimerization domain of the female germline-specific tumor suppressor protein gld-1 (PDB: 3kbl; confidence 59%; identity 27%).

## 4. Discussion

The family *Bunyaviridae* currently has 530 putative members [1]. These members are either classified in the five recognized bunyaviral genera *Hantavirus*, *Nairovirus*, *Orthobunyavirus*, *Phlebovirus*, and *Tospovirus*, or remain to be classified into these existing or novel genera. Until recently, bunyaviral classification predominantly relied on antigenic relationships as genomic sequence information on individual bunyaviruses was scarce [1]. Classical and next-generation sequencing is now increasingly applied to historical isolates of presumed or newly discovered putative bunyaviruses [6,7,8,9,10,11,12,13,14,15,16,17,18,19,20,21,22,23,24,25,26,27,28,29,30,31,32,33,34,35,36,37,38,39,40,41,42]. These studies demonstrated that bunyaviral diversity is far broader than previously appreciated, probably necessitating the establishment of additional bunyaviral genera [22,23,24]. Other studies revealed that arenaviruses, emaraviruses, tenuiviruses, and Mourilyan virus may have to be included among bunyaviruses despite having genomes with more or less than the bunyavirus-typical three segments [119,120,121,122,123,124,125,126]. Finally, although sequencing of putative bunyaviruses by and large confirmed historical antigenic classifications, several bunyaviruses were assigned to wrong genera [14,25,26,27,28,29,30,31,32,33,34].

Broadening the bunyaviral sequence space to encompass as many bunyaviruses and bunya-like viruses as possible is necessary to elucidate their true phylogenetic relationships and to establish a modern comprehensive bunyaviral taxonomy that adequately reflects evolution. Here we reported 23 bunyaviral genome sequences (Table 1) with the goal of understanding the relationships of nairoviruses and nairo-like viruses—a group of bunyaviruses for which almost no sequence information was available until very recently [29,42]. While we confirmed the overall monophyly of the current genus *Nairovirus*, our results indicated that (a) novel species will have to be established; (b) that some nairoviruses will have to be moved between species; (c) that a long-thought phlebovirus is actually a nairovirus; and (d) that several newly discovered bunyaviruses do not directly fall into the nairovirus clade but are nevertheless more closely related to nairoviruses than to other bunyaviruses (Figure 1, Figure 2 and Figure 3). We propose here that in the absence of at least coding-complete genomic information, “bunyaviruses” should not be classified into bunyaviral taxa but rather be seen as putative members of the family *Bunyaviridae*. Applying this stringent criterium to the list of “nairoviruses” (Table S1), we propose a new taxonomic organization for the genus *Nairovirus* in Table 2. This organization is overall in line with a similar, very recent, proposal by Walker *et al.* [42].

Coding-complete genome sequences of at least five additional nairoviruses (Burana virus, Caspiyi virus, Chim virus, Geran virus, and Tamdy virus) have been determined but are not yet available for analysis [30,31,32,33,34] (Table S1). Therefore, the taxonomy proposed in Table 2 should be considered preliminary until these sequences become available and incorporated. In particular, the Burana virus and Tamdy virus sequences may help to further refine the Burana and Qalyub genogroups.

Genomic reassortment (*i.e.*, the relatively free swapping of S, M, and L segments, between taxonomically diverse bunyaviruses simultaneously infecting the same host and consequently resulting in novel viruses) has been described for orthobunyaviruses, phleboviruses, and tospoviruses [127]. Interestingly, our analysis did not reveal any signs of inter-nairovirus reassortment. This result suggests that distinct nairoviruses may rarely have the opportunity in nature to infect the same host at the same time or that molecular-biological constraints prevent inter-nairovirus reassortment. *In vitro* experiments should be performed to evaluate these hypotheses.

Two recent studies on arthropod samples revealed the existence of at least five bunyaviruses (SWSV-1, Shāyáng spider virus 1, SBV, Wǔhàn millipede virus 2, and XSV) that appeared to be closely related to nairoviruses [37,41]. Interestingly, SBV and Wǔhàn millipede virus 2 genomes were found to consist of only two segments, rather than the bunyavirus/nairovirus-typical three segments. Our phylogenetic analyses (Figure 1, Figure 2 and Figure 3 and Figure 8) indicate that all five viruses should not be classified in the genus *Nairovirus*, but confirm that these viruses are more closely related to nairoviruses than to all other bunyaviruses. To further resolve phylogenetic placement of these five viruses, we performed PAirwise Sequence Comparison (PASC) of bunyavirus sequences using the National Center for Biotechnology Information’s (NCBIs) PASC tool. Histograms from this analysis demonstrates the distribution of the number of virus genome pairs at each identity percentage. Histogram peaks and valleys can be used to differentiate taxon ranks and to establish taxon demarcation criteria using objective criteria [128,129]. A preliminary PASC analysis with representative bunyaviral genomes indicate that cut-offs of ≈26% and 31%–34% identity for the M and L segments, respectively, ought to be used to uphold the current bunyaviral division into *Hantavirus*, *Nairovirus*, *Orthobunyavirus*, *Phlebovirus*, and *Tospovirus* genera (data not shown). Using these cut-offs, PASC confirms the monophyly of the genus *Nairovirus* as outlined in Table 2 and indicates the need to establish four nairovirus-like genera for (1) SBV; (2) SWSV-1 and XSV; (3) Shāyáng spider virus 1; and (4) Wǔhàn millipede virus 2 (Figure 1, Figure 2 and Figure 3; Figure 8: nairoviruses in blue and nairo-like viruses in purple). Interestingly, phylogenetic analyses also indicate that the bisegmented arenaviruses, currently not part of the *Bunyaviridae*, are more related to these viruses than they are to other bunyaviruses, suggesting the need to establish an arenavirus/nairovirus supergroup within the family (Figure 8).

## Figures and Tables

**Table 1:** Viruses sequenced for this study. NCR, noncoding regions; RSFSR, Russian Soviet Federated Socialist Republic; USSR, United Soviet Socialist Republic.

Virus Name (Abbreviation)	Strain Designation	Source	Date; Place of Isolation	Ref.	BioSampleID GenBank Accession Numbers	L 5′ NCR	L 3′ NCR	M 5′ NCR	M 3′ NCR	S 5′ NCR	S 3′ NCR
Abu Hammad virus (AHV)	Eg ArT 1194	Ticks (*Argas hermanni*) collected from pigeon	7 June 1971; Abu Hammad, al-Sharqia Governorate, Egypt	[43]	Re-sequenced [42]: SAMN04530531	Yes	Yes	Yes	Yes	Yes	Yes
					S: KU925436						
					M: KU925435						
					L: KU925434						
Abu Mina virus (AMV)	Eg An 4996-63	European turtle dove (*Streptopelia turtur*) and associated ticks (*Argas streptopelia*)	1 May 1963; Abu Mina, Matrouh Governorate, Egypt	[43]	Newly sequenced: SAMN04530533	Yes	Yes	Yes	Yes	Yes	Yes
					S: KU925439						
					M: KU925438						
					L: KU925437						
Avalon virus (AVAV)	Brest/Ar T261	Ticks (*Ixodes uriae*)	1979; Brittany, France	[44]	Newly sequenced: SAMN04530548	Yes	Yes	Yes	Yes	Yes	Yes
					S: KU925445						
					M: KU925444						
					L: KU925443						
Avalon virus (AVAV)	CanAr 173	Ticks (*Ixodes uriae*) from European herring gull (*Larus argentatus*)	31 July 1972; Great Island, Newfoundland and Labrador, Canada	[45]	Re-sequenced [42]: SAMN04530547	Yes	Yes	Yes	Yes	Yes	Yes
					S: KU925442						
					M: KU925441						
					L: KU925440						
Bandia virus (BDAV)	IPD/A 611	Rodent (*Mastomys* sp.) and ticks (*Ornithodoros sonrai*) collected from rodent burrow	26 February 1965; Bandia Forest, Thiès Region, Senegal	[46]	Re-sequenced [42]: SAMN04530545	No	No	No	No	No	No
					S: KU925448						
					M: KU925447						
					L: KU925446						
Clo Mor virus (C[L]MV)	ScotAr 7	Ticks (*Ixodes uriae*) in nesting sites of common murees (*Uria aalge*)	15 June 1973; Clo Mor, Cape Wrath, Scotland, UK	[45]	Newly sequenced: SAMN04530553	No	No	No	No	No	No
					S: KU925451						
					M: KU925450						
					L: KU925449						
Dera Ghazi Khan virus (DGKV)	JD 254	Ticks (*Hyalomma dromedarii*) collected from a camelid	4 April 1966; Dera Ghazi Khan District, Punjab Province, Pakistan	[47]	Re-sequenced [42]: SAMN04530534	Yes	Yes	Yes	Yes	Yes	Yes
					S: KU925454						
					M: KU925453						
					L: KU925452						
Dugbe virus (DUGV)	IbAr 1792	Ticks (*Amblyomma variegatum*) collected from cattle	14 October 1964; Ibadan, Oyo State, Nigeria	[48]	Newly sequenced: SAMN04530543	Yes	Yes	Yes	Yes	Yes	Yes
					S: KU925457						
					M: KU925456						
					L: KU925455						
Erve virus (ERVEV)	Brest/An 221 (TVP21049)	Greater white-toothed shrew (*Crocidura russula*)	5 May 1982; Saulges, Mayenne Départment, France	[49]	Re-sequenced [42]: SAMN04530552	No	No	No	No	Yes	Yes
					S: KU925460						
					M: KU925459						
					L: KU925458						
Farallon virus (FARV)	Cal Ar846	Ticks (*Carios denmarki*)	20 July 1965; Farallon Islands, California, USA	[50]	Re-sequenced [42]: SAMN04530536	Yes	Yes	Yes	Yes	Yes	Yes
					S: KU925463						
					M: KU925462						
					L: KU925461						
Ganjam virus (GANV)	G 619 (TVP20486)	Ticks (*Haemaphysalis intermedia*) collected from a domestic goat	6 November 1954; Bhanjanagar, Ganjam District, Orissa, India	[51]	Re-sequenced (Yadav *et al.*, unpublished) SAMN04530544	No	Yes	No	No	No	No
					S: KU925466						
					M: KU925465						
					L: KU925464						
Great Saltee virus (GRSV)	RML 59972	Ticks (*Carios maritimus*) collected from a seabird nest	1972; Great Saltee Island, County Wexford, Ireland	[52]	Newly sequenced: SAMN04530537	Yes	Yes	Yes	Yes	Yes	Yes
					S: KU925469						
					M: KU925468						
					L: KU925467						
Hughes virus (HUGV)	G2126	Ticks (*Carios denmarki*)	January, 1962; Bush Key, Dry Tortugas, Florida, USA	[53,54]	Newly sequenced: SAMN04530538	Yes	Yes	Yes	Yes	Yes	Yes
					S: KU925472						
					M: KU925471						
					L: KU925470						
Punta Salinas virus (PSV)	Cal Ar888	Ticks (*Carios amblus*)	14 October 1967; Punta Salinas, Huaura Province, Lima Region, Peru	[55]	Re-sequenced [42]: SAMN04530539	No	No	No	No	No	No
					S: KU925475						
					M: KU925474						
					L: KU925473						
Qalyub virus (QYBV)	Eg Ar 370	Ticks (*Carios erraticus*) collected from a rat nest	28 August 1952; Qalyub, al-Qalyubiyah Governorate, Egypt (British Protectorate)	[56]	Re-sequenced [42]: SAMN04530546	Yes	Yes	Yes	Yes	Yes	Yes
					S: KU925478						
					M: KU925477						
					L: KU925476						
Raza virus (RAZAV)	829	Ticks (*Carios denmarki*)	20 May 1962; Raza Island, Baja California, Mexico	[57]	Newly sequenced: SAMN04530540	Yes	Yes	Yes	No	Yes	Yes
					S: KU925481						
					M: KU925480						
					L: KU925479						
Sakhalin virus (SAKV)	LEIV-71C	Ticks (*Ixodes uriae*) collected from nesting sites of common murees (*Uria aalge*)	21 November 1969; Tyuleniy Island, Sea of Okhotsk, Sakhalin Oblast, RSFSR, USSR	[58]	Newly sequenced: SAMN04530549	No	No	No	Yes	No	No
					S: KU925484						
					M: KU925483						
					L: KU925482						
Sapphire II virus (SAPV)	RML 52323-14	Ticks (*Argas cooleyi*) collected from a cliff swallow nest	August 1969; Garza County, Texas, USA	[59]	Newly sequenced: SAMN04530535	Yes	Yes	Yes	Yes	Yes	Yes
					S: KU925487						
					M: KU925486						
					L: KU925485						
Soldado virus (SOLV)	TRVL 52214	Ticks (*Carios capensis*)	16 June 1963; Soldado Rock, Trinidad and Tobago	[60]	Newly sequenced: SAMN04530541	Yes	Yes	Yes	Yes	Yes	Yes
					S: KU925490						
					M: KU925489						
					L: KU925488						
Taggert virus (TAGV)	Ml14850	Ticks (*Ixodes uriae*) from seabird rookery	1 January 1972; Macquarie Island, Tasmania, Australia	[61]	Re-sequenced [42]: SAMN04530550	No	Yes	No	Yes	Yes	Yes
					S: KU925493						
					M: KU925492						
					L: KU925491						
Tillamook virus (TILLV)	RML 86	Ticks (*Ixodes uriae*)	1970; Oregon, USA	[62]	Newly sequenced: SAMN04530551	Yes	Yes	Yes	Yes	Yes	Yes
					S: KU925496						
					M: KU925495						
					L: KU925494						
Tunis virus (TUNV)	Brest/Ar/T2756	Ticks (*Argas hermanni*)	October 1989; El Kef, Kef Governorate, Tunisia	[63]	Newly sequenced: SAMN04530532	Yes	Yes	Yes	Yes	Yes	Yes
					S: KU925499						
					M: KU925498						
					L: KU925497						
Zirqa virus (ZIRV)	POR7866	Ticks (*Carios muesebecki*)	2 November 1969; Zirku (Zirqa/Zarrakuh) Island, Abu Dhabi, United Arab Emirates	[64]	Newly sequenced: SAMN04530542	Yes	Yes	No	Yes	No	Yes
					S: KU925502						
					M: KU925501						
					L: KU925500						

**Table 2:** Proposed new taxonomy of the genus *Nairovirus* based on genomic data. Viruses mentioned in Table S1 but not here ought to be considered putative nairoviruses that based on current data cannot/should not be classified. Proposed new taxa are highlighted in red and placed in quotation marks.

Species	Virus Members
“*Burana Nairovirus*”	Huángpí tick virus 1 (HTV-1)
	Tǎchéng tick virus 1 (TTV-1)
	Wēnzhōu tick virus (WTV)
*Crimean-Congo hemorrhagic fever nairovirus*	Crimean-Congo hemorrhagic fever virus (CCHFV)
	Abu Hammad virus (AHV) including Tunis isolate
*Dera Ghazi Khan nairovirus*	Abu Mina virus (AMV)
	Dera Ghazi Khan virus (DGKV)
	Sapphire II virus (SAPV)
*Dugbe nairovirus*	Dugbe virus (DUGV)
	Kupe virus (KUPEV)
*“Hazara nairovirus*”	Hazara virus (HAZV)
	Tofla virus (TFLV)
*Hughes nairovirus*	Caspiy virus (CASV)
	Farallon virus (FARV)
	Great Saltee virus (GRSV)
	Hughes virus (HUGV)
	Punta Salinas virus (PSV)
	Raza virus (RAZAV)
	Soldado virus (SOLV)
	Zirqa virus (ZIRV)
“*Keterah nairovirus*”	Gossas virus (GOSV)
	Issyk-kul virus (ISKV)
	Keterah virus (KTRV) including soft tick isolate
	Uzun-Agach virus (UZAV)
“*Kasokero nairovirus*”	Kasokero virus (KAS(O)V)
	Leopards Hill virus (LPHV)
	Yogue virus (YOGV)
“*Nairobi sheep disease virus nairovirus*”	Ganjam virus (GANV)
	Nairobi sheep disease virus (NSDV) including Ganjam isolate
*Qalyub nairovirus*	Bandia virus (BDAV)
	Qalyub virus (QYBV)
*Sakhalin nairovirus*	Avalon virus (AVAV)
	Clo Mor virus (C(L)MV)
	Sakhalin virus (SAKV)
	Taggert virus (TAGB)
	Tillamook virus (TILLV)
*Thiafora nairovirus*	Erve virus (ERVEV)
	Thiafora virus (TFAV)

## Supplementary Materials

The following are available online at www.mdpi.com/link, Table S1: All known and putative nairoviruses and nairo-like viruses and their deduced relationships prior to this study; Table S2: M-segment polyprotein descriptions. Figures S1 through S5: conserved features of nairovirus RNA-dependent RNA polymerases (Ls).
