# Supplementary material for: Genomic Characterization of the Genus Nairovirus (Family Bunyaviridae)
Source: Viruses. 2016 Jun 10;8(6):164. doi: 10.3390/v8060164 (PMC4926184; doi:10.3390/v8060164)
Supplement: Supplementary file 1 [file viruses-08-00164-s001.docx]

Supplementary material: Genomic Characterization of the Genus *Nairovirus* (Family *Bunyaviridae*)

Jens H. Kuhn ^1^, Michael R. Wiley ^2^, Sergio E. Rodriguez ^3^, Yīmíng Bào ^4^, Karla Prieto ^2^,
Amelia P. A. Travassos da Rosa ^3^, Hilda Guzman ^3^, Nazir Savji ^5^, Jason T. Ladner ^2^,
Robert B. Tesh ^3^, Jiro Wada ^1^, Peter B. Jahrling ^1^, Dennis A. Bente ^3^ and Gustavo Palacios ^2,^*


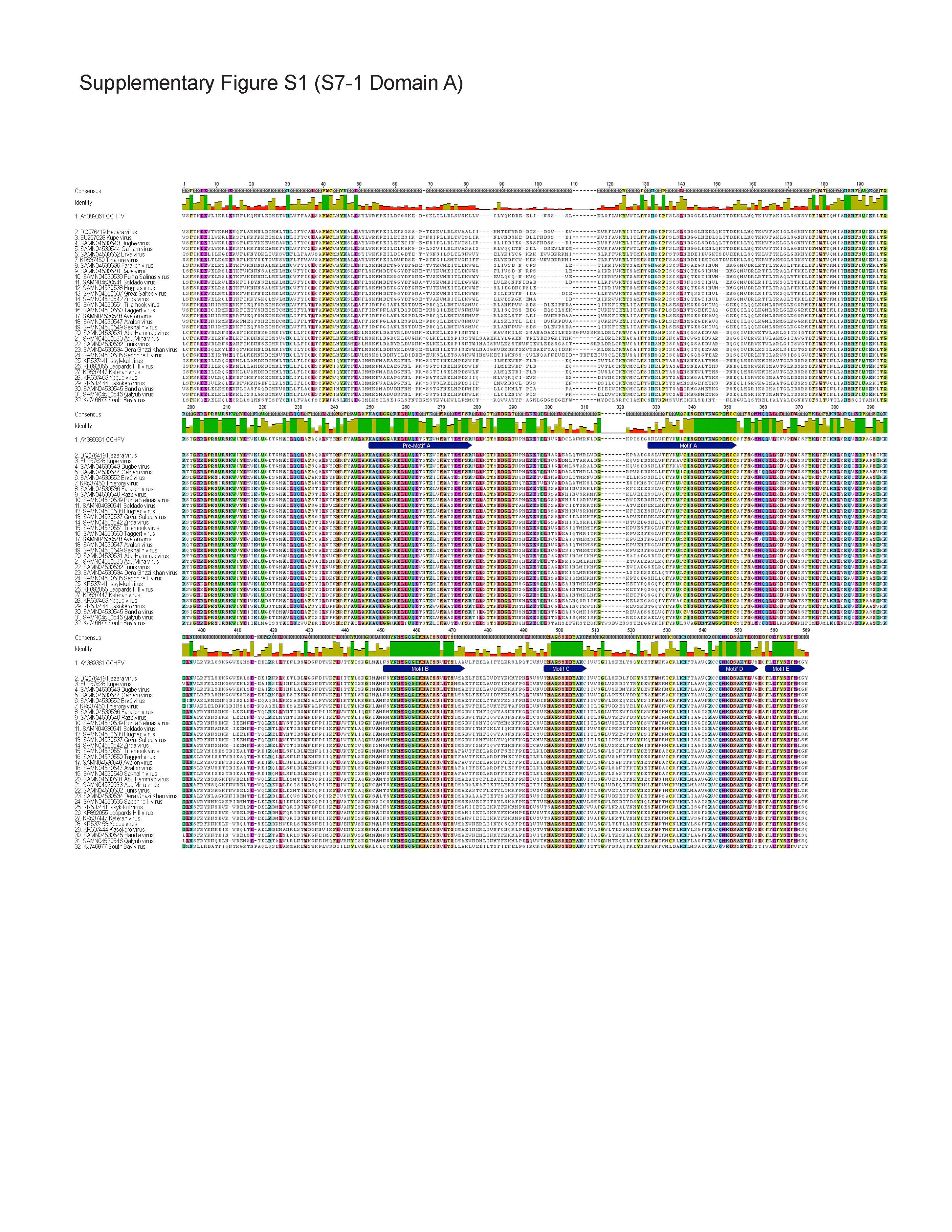


**Figure S1**: S7-1 Domain A


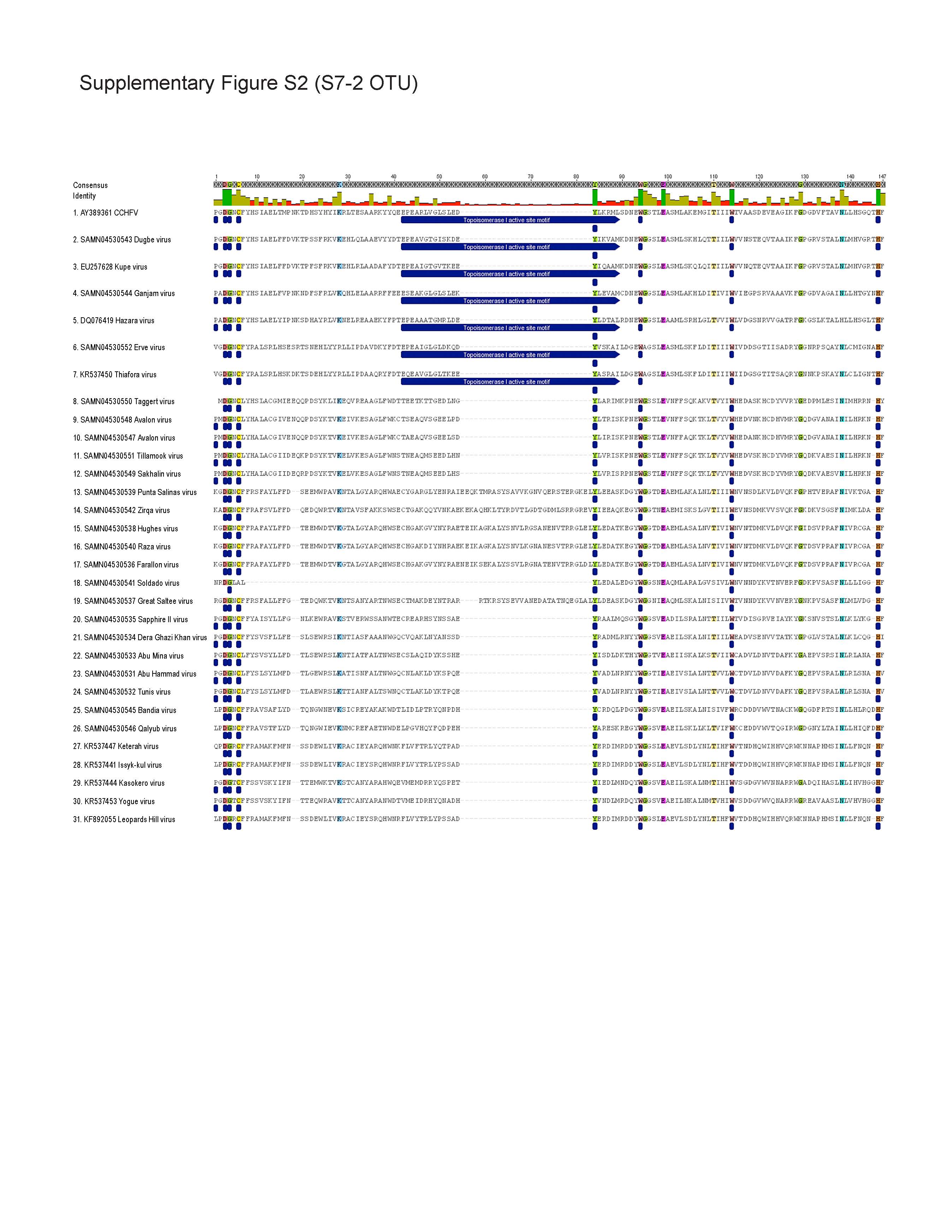


**Figure S2:** S7-2 OTU


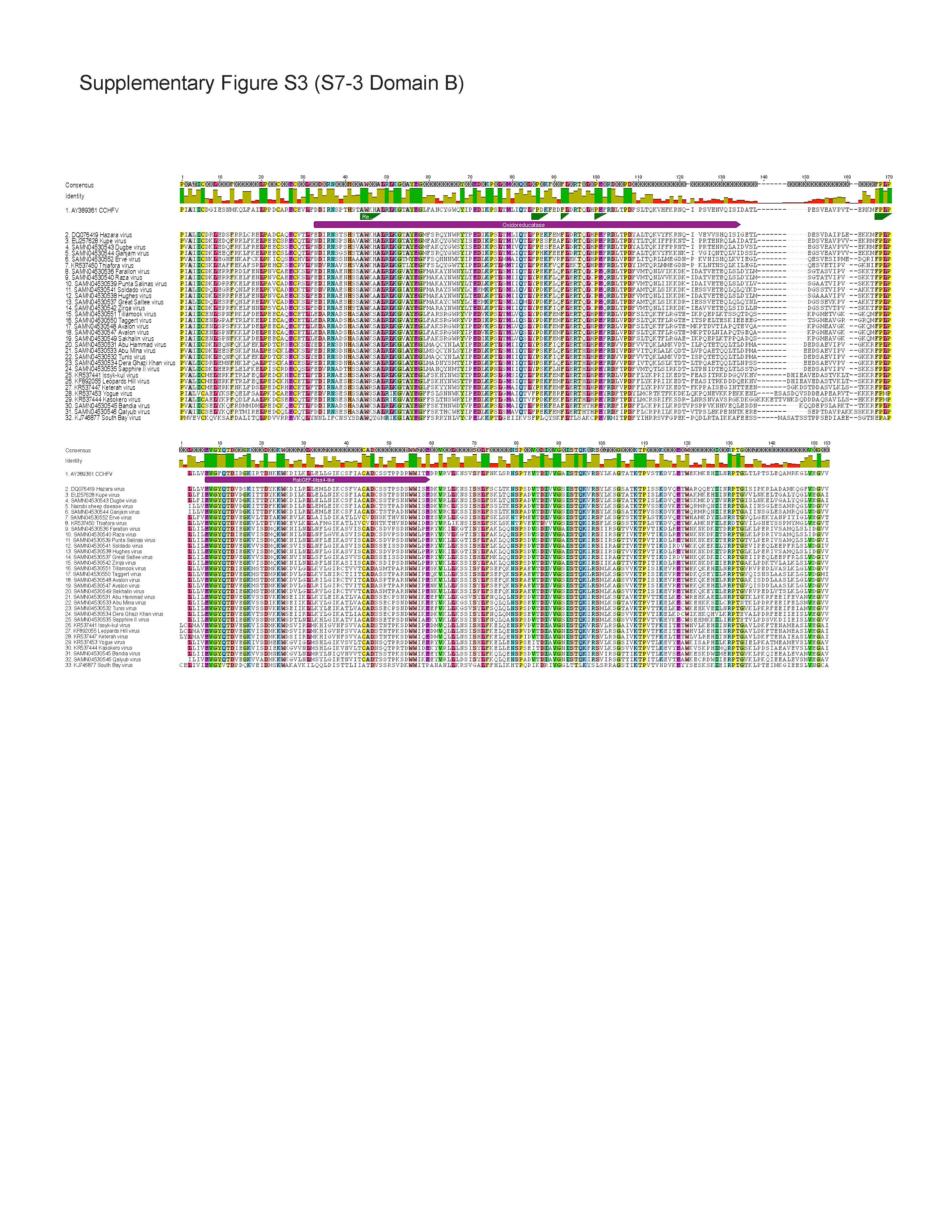


**Figure S3:** S7-3 Domain B


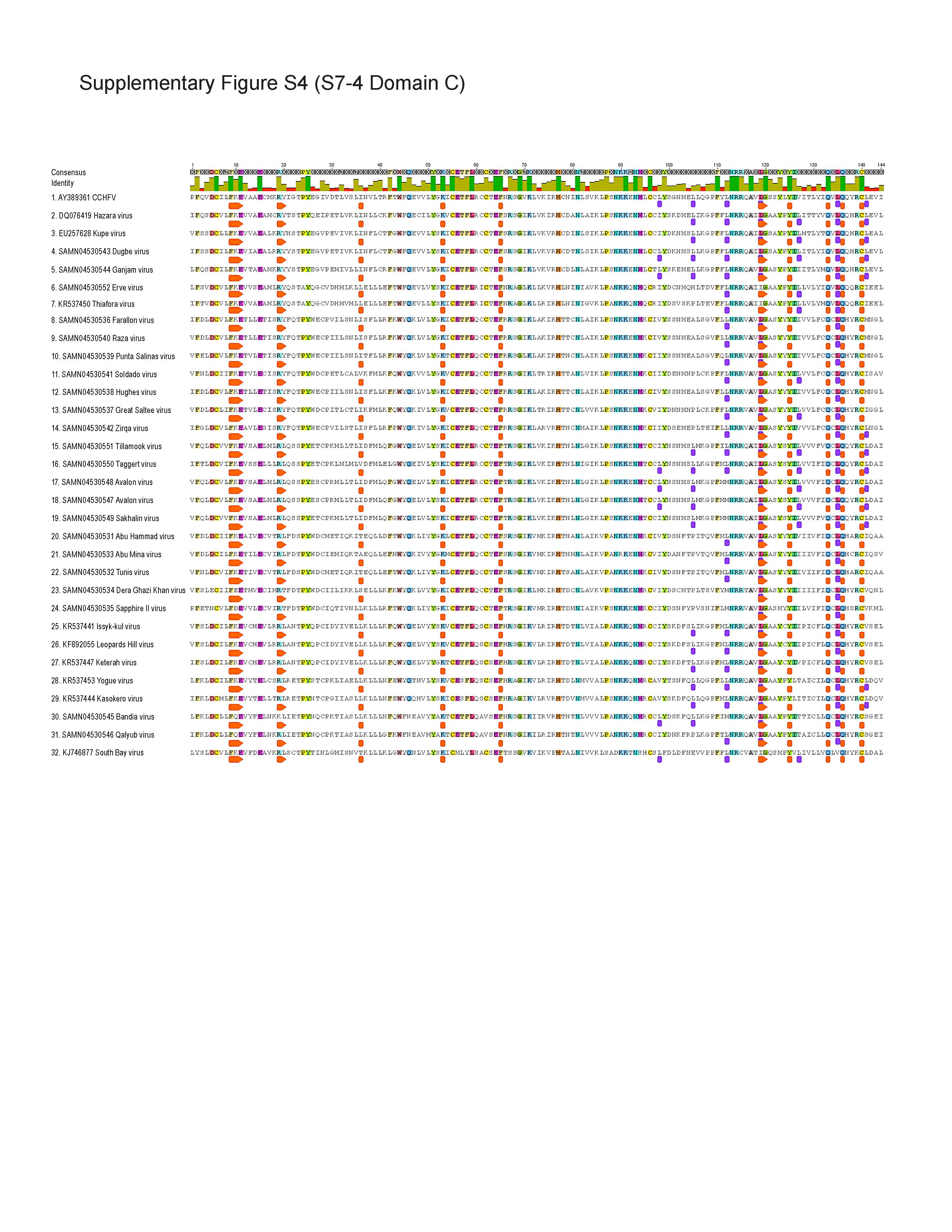


**Figure S4:** S7-4 Domain C

**
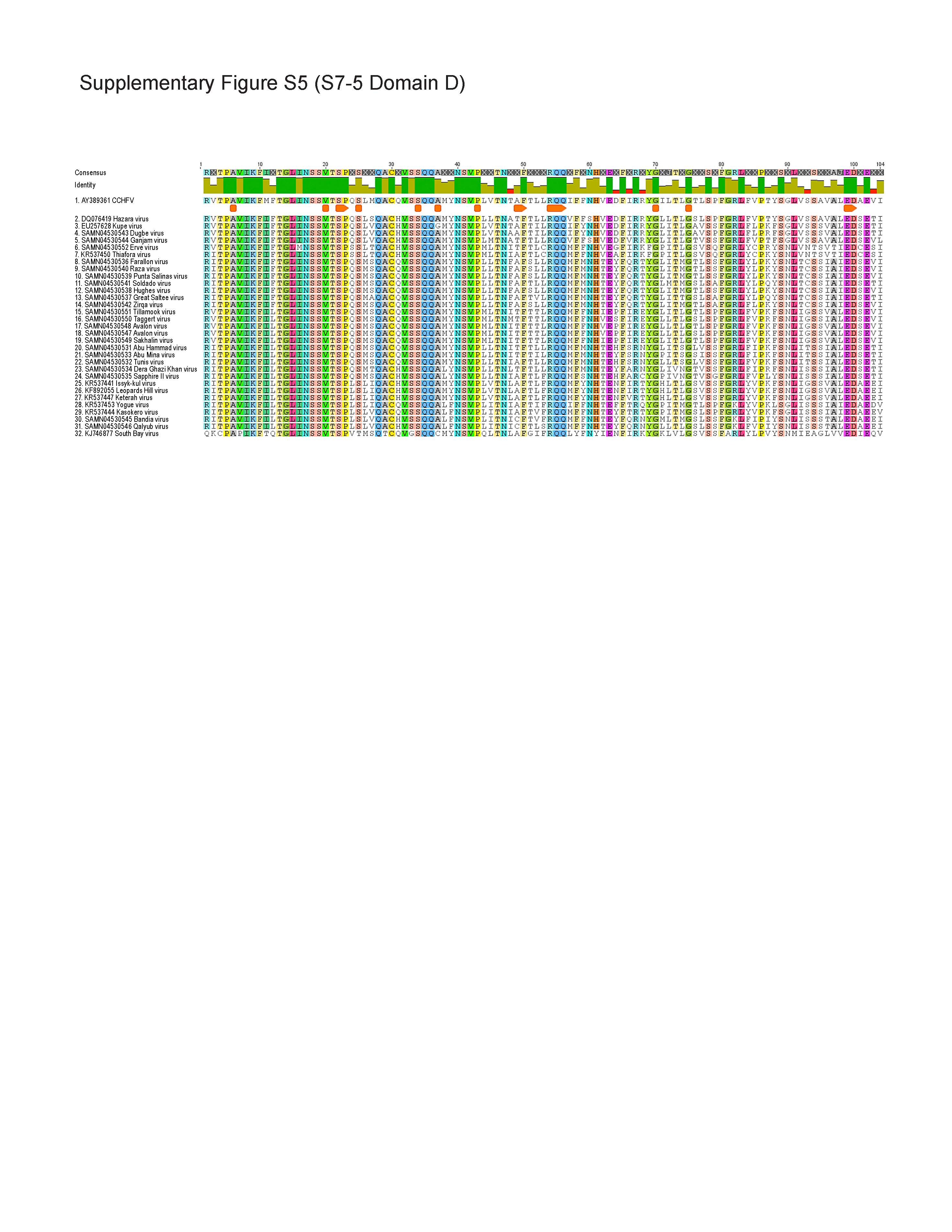
**

**Figure S5:** S7-5 Domain D

**Table S1.** Nairovirus and nairo-like viruses and their deduced relationships prior to this study (based in part on [[1‒23](#_ENREF_1)]). a Genomic segments are representative complete or coding-complete sequences. NK, not known; NR, not recorded; TBD, to be determined.

| **Nairovirus Species and Virus Members (Abbreviations)** | **Virus Vector(s)** | **Virus Vertebrate Host(s)** | **Human infections** | **Genomic Segment Accession Numbers (RefSeq/GenBank)^a^** |
| --- | --- | --- | --- | --- |
| **Species *Crimean-Congo hemorrhagic fever nairovirus*** |  |  |  |  |
| Crimean-Congo hemorrhagic fever virus (CCHFV) | Biting midges (*Culicoides* sp.)  Ticks (*Alveonasus lahorensis*, *Argas persicus*, *Amblyomma* spp., *Dermacentor dagestanicus*, *D. marginatus*, *Haemaphysalis punctata*, *Hyalomma anatolicum, H. asiaticum, H. detritum, H. excavatum*, *H. impeltatum*, *H. impressum*, *H. lusitanicum*, *H. marginatum*, *H. nitidum*, *H. punctatum*, *H. truncatum*, *Ixodes ricinus*, *Rhipicephalus annulatus*, *R.* *bursa*, *R. decoloratus*, *R. geigyi*, *R. microplus*, *R. pulchellus, R.* *pumilio*, *R.* *rossicus*, *R.* *sangineus*, *R. turanicus*) | Birds, camelids, cattle, eulipotyphla, humans, lagomorphs, rodents, sheep | Yes: Crimean-Congo hemorrhagic fever (frequently lethal) | S: NC_005302.1  M: NC_005300.2  L: NC_005301.3 |
| Hazara virus (HAZV) | Ticks (*Ixodes redikorzevi*) | Royle’s mountain voles (*Alticola roylei*)? | NR | S: KP406725.1  M: KP406724.1  L: KP406723.1 |
| Tofla virus (TFLV) | Ticks (*Heamaphysalis flava* and *Heamaphysalis formsensis****)*** | Rodents? | NR | S: LC008510.1  M: LC008511.1  L: LC008512.1 |

| **Species *Dera Ghazi Khan nairovirus*** |  |  |  |  |
| --- | --- | --- | --- | --- |
| Abu Hammad virus (AHV) | Ticks (*Argas* *hermanni*) | Pigeons? | NR | This study and [[23](#_ENREF_23)]:  S. KU343144.1  M: KU343143.1  L: KU343142.1 |
| Abu Mina virus (AMV) | Ticks (*Argas* *streptopelia*) | European turtle doves (*Streptopelia turtur*)? | NR | This study |
| Dera Ghazi Khan virus (DGKV) | Ticks (*Hyalomma dromedarii*) | Camelids? | NR | This study and [[23](#_ENREF_23)]:  S: KU343153.1  M: KU343152.1  L: KU343151.1 |
| Kao Shuan virus (KSV) | Ticks (*Argas* *robertsi*) | Night herons (*Nycticorax nycticorax*)? | NR | TBD |
| Pathum Thani virus (PTHV) | Ticks (*Argas* *robertsi*) | Open-billed storks (*Anastomus ascitans*)? | NR | TBD |
| Pretoria virus (PREV) | Ticks (*Argas* *africolumbae*) | Cape rock pigeons (*Columba guinea phaeonata*)?) | NR | TBD |
| Sapphire II virus (SAPV) | Ticks (*Argas cooley*) | Seabirds? | NR | This study and [[23](#_ENREF_23)]:  S: KU343165.1  M: KU343164.1  L: KU343163.1 |
| **Species *Dugbe nairovirus*** |  |  |  |  |
| Dugbe virus (DUGV) | Biting midges (*Culicoides* sp.)  Ticks (*Amblyomma variegatum*, *A. lepidum*, *Hyalomma truncatum*, *H. rufipes*, *Ixodes* sp., *Rhipicephalus decoloratus*,) | Cattle, giant pouched rats (*Cricetomys gambianus*) | Yes (rare): diarrhea, fever, headache, maculopapular rash, nausea, vomiting (non-lethal) | S: NC_004157.1  M: NC_004158.1  L: NC_004159.1 |
| Ganjam virus (GANV) | Mosquitoes (*Culex vishnui*)  Ticks (*Haemaphysalis* *intermedia*, *H.* *wellingtoni*, *Rhipicephalus haemaphysaloides*) | Domestic sheep and goats | Yes (rare): fever (non-lethal) | Yadav *et al*. and this study:  S: AF504294.1  M: EU697950.1  L: EU697949.1 |
| Kupe virus (KUPEV) | Ticks (*Amblyomma gemma*, *Rhipicephalus pulchellus*) | Livestock? | NR | S: EU257626.1  M: EU257627.1  L: EU257628.1 |
| Nairobi sheep disease virus (NSDV) | Ticks (*Amblyomma variegatum*, *Haemaphysalis intermedia*, *Rhipicephalus* *appendiculatis*, *R. pulchellus*, *R. simus*) | Domestic sheep and goats | Yes (rare): abdominal pain, fever, headache, myalgia (non-lethal) | S: KM464724.1  M: KM464725.1  L: KM464726.1 |
| **Species *Hughes nairovirus*** |  |  |  |  |
| Caspiy virus (CASV) | Ticks (*Carios capensis*) | Common terns (*Sterna hirundo*), European herring gulls (*Larus argentatus*) | NR | Not yet deposited |
| Elliðaey virus ELL 81-3b | Ticks (*Ixodes uriae*) | Atlantic puffins (*Fratercula arctica*)? | NR | TBD |
| Farallon virus (FARV) | Ticks (*Carios denmarki*) | Western gulls (*Larus occidentalis*)? | NR | This study and [[23](#_ENREF_23)]:  S: KU343156.1  M: KU343155.1  L: KU343154.1 |
| Fraser Point virus (FPV) | NK | NK | NR | TBD |
| Foula virus F 80-1 | Ticks (*Ixodes uriae*) | Common murees (*Uria aalge*)? | NR | TBD |
| Great Saltee virus (GRSV) | Ticks (*Carios maritimus*, *Ixodes uriae*) | Black-legged kittiwakes (*Rissa tridactyla*), European shags (*Phalacrocorax aristotelis*), Razorbills (*Alca torda*)? | NR | This study |
| Grímsey virus GRIMS82-1b | Ticks (*Ixodes uriae*) | Atlantic puffins (*Fratercula arctica*)? | NR | TBD |
| Hughes virus (HUGV) | Ticks (*Carios denmarki*) | Brown noddies (*Anous stolidus*), sooty terns (*Sterna fuscata*)? | NR | This study |
| Inner Farne Island virus IF 80-3, IF80-4 | Ticks (*Ixodes uriae*) | Common murees (*Uria aalge*)? | NR | TBD |
| Island of May virus IM81 | Ticks (*Ixodes uriae*) | Common murees (*Uria aalge*)? | NR | TBD |
| Mykines virus M82-2 | Ticks (*Ixodes uriae*) | Atlantic puffins (*Fratercula arctica*)? | NR | TBD |
| Puffin Island virus (PIV) | Ticks (*Carios maritimus*) | Seabirds? | NR | TBD |
| Punta Salinas virus (PSV) | Ticks (*Argas arboreus*, *Carios amblus*) | Guanay cormorants (*Phalacrocorax bougainvillii*), Peruvian boobies (*Sula variegata*)? | NR | This study and [[23](#_ENREF_23)]:  S: KU343159.1  M: KU343158.1  L: KU343157.1 |
| Raza virus (RAZAV) | Ticks (*Carios denmarki*) | Seabirds? | NR | This study |
| Soldado virus (SOLV) | Ticks (*Amblyomma loculosum*, *Carios capensis*, *C. denmarki*) | Brown noddies (*Anous stolidus*), Cape cormorants (*Phalacrocorax capensis*), great cormorants (*Phalacrocorax carbo*), grey-headed gulls (*Chroicocephalus cirrocephalus*), long-tailed cormorants (*Phalacrocorax africanus*), masked boobies (*Sula dactylatra*), sooty terns (*Sterna fuscata*)? | NR | This study |
| Zirqa virus (ZIRV) | Ticks (*Carios* *muesebecki*) | Ospreys (*Pandion haliaetus*), Socotra cormorants (*Phalacrocorax nigrogularis*)? | NR | This study and [[23](#_ENREF_23)]:  S: KU343171.1  M: KU343170.1  L: KU343169.1 |
| **Species *Qalyub nairovirus*** |  |  |  |  |
| Bakel virus (BAKV) | Ticks (*Ornithodoros sonrai*) | NK | NR | TBD |
| Bandia virus (BDAV) | Ticks (*Ornithodoros sonrai*) | Multimammate rats  (*Mastomys* sp.) | NR | This study and [[23](#_ENREF_23)]:  S: KU343150.1  M: KU343149.1  L: KU343148.1 |
| Chim virus (CHIMV) | Ticks (*Hyalomma asiaticum*, *Ornithodoros papillipes, O. tartakovskii*, *Rhipicephalus turanicus*) | Great gerbils (*Rhombomys opimus*)? | NR | Not yet deposited |
| Geran virus (GERV/GRNV) | Ticks (Ornithodoros verrucosus) | Libyan jirds (*Meriones lybicus*)? | NR | Not yet deposited |
| Omo virus (OMOV) | NK | Reddish-white mastomys (*Mastomys erythroleucus*) | NR | TBD |
| Qalyub virus (QYBV) | Ticks (*Carios erraticus*) | African arvicanthis (*Arvicanthis niloticus*), camelids, cattle, pigs, dogs, donkeys | NR | This study and [[23](#_ENREF_23)]:  S: KU343162.1  M: KU343161.1  L: KU343160.1 |
| **Species *Sakhalin nairovirus*** |  |  |  |  |
| Avalon virus (AVAV) = Paramushir virus (PRMV)? | Ticks (*Ixodes signatus*, *I.* *uriae*) | Atlantic puffins (*Fratercula arctica*), European herring gulls (*Larus argentatus*), Leach's storm petrels (*Oceanodroma leucorhoa*)? | NR | This study and [[23](#_ENREF_23)]:  S: KU343147.1  M: KU343146.1  L: KU343145.1 |
| Clo Mor virus (C[L]MV) | Ticks (*Ixodes* *uriae*) | Common murees (*Uria aalge*)? | NR | This study and [[23](#_ENREF_23)]:  S: KU343141.1  M: KU343140.1  L: KU343139.1 |
| Finch Creek virus (FINCV) | Ticks (*Ixodes uriae*) | Penguins? | NR | TBD |
| Kachemak Bay virus (KBV) | Ticks (*Ixodes signatus*) | Common murees (*Uria aalge*)? | NR | TBD |
| Sakhalin virus (SAKV) | Ticks (*Ixodes putus*, *I. signatus*, *I. uriae*) | Black-legged kittiwakes (*Rissa tridactyla*), common murees (*Uria aalge*), Northern fulmars (*Fulmaris flacialis*), red-necked phalaropes (*Phalaropus lobatus*), tufted puffins (*Lunda cirrhata*)? | NR | This study |
| Taggert virus (TAGB) | Ticks (*Ixodes* *uriae*) | Macaroni penguins (*Eudyptes chrysolophus*)? | NR | This study and [[23](#_ENREF_23)]:  S: KU343168.1  M: KU343167.1  L: KU343166.1 |
| Tillamook virus (TILLV) | Ticks (*Ixodes uriae*) | Seabirds? | NR | This study |
| **Species *Thiafora nairovirus*** |  |  |  |  |
| Erve virus (ERVEV) | NK | Greater white-toothed shrews (*Crocidura russula*) | Possibly: thunderclap headaches (non-lethal)? | S: JF911699.1  M: JF911698.1  L: JF911697.1 |
| Thiafora virus (TFAV) | NK | White-toothed shrews (*Crocidura* sp.) | NR | Coding-complete:  S: KR537452.1  M: KR537451.1  L: KR537450.1 |
| **Putative species “*Ahun nairovirus*”** |  |  |  |  |
| Ahun virus | NK | Common pipistrelles (*Pipistrellus pipistrellus*), whiskered myotis (*Myotis mystacinus*) | NR | TBD |

| **Putative species “*Issyk-kul nairovirus/Keterah nairovirus*”** |  |  |  |  |
| --- | --- | --- | --- | --- |
| Garm virus | NK | Common redstarts (*Phoenicurus phoenicurus*) | NR | TBD |
| Gossas virus (GOSV) | NK | Free-tailed bats (*Tadarida* sp.) | NR | Coding-complete:  S: KR534876.1  M: KR534877.1  L: KR534878.1 |
| Issyk-kul virus (ISKV) | Biting midges (*Culicoides schultzei*)  Horseflies (*Tabanus agrestis*)  Mosquitoes (*Aedes caspius*, *Anopheles hyrcanus*)  Ticks (*Argas vespertilionis, A. pusillus*, *Ixodes vespertilionis*) | Humans  Bats: noctules (*Nyctalus noctula*), lesser mouse-eared myotis (*Myotis blythii*), common serotines (*Eptesicus serotinus*), particolored bats (*Vespertilio murinus*), common pipistrelles (*Pipistrellus pipistrellus*), greater horseshoe bats (*Rhinolophus ferrumequinum*)  Birds: Spanish sparrows (*Passer hispaniolensis*), white wagtails (*Motacilla alba*), grey wagtails (*Motacilla cinerea*), common redstarts (*Phoenicurus phoenicurus*), house swallows (*Hirundo rustica*), wrynecks (*Jynx torquilla*), common kingfishers (*Alcedo atthis*) | Yes (rare): cough, fever, headache, nausea (non-lethal) | Complete:  S: KF892057.1  M: KF892056.1  L: KF892055.1 |
| Keterah virus (KTRV) | Ticks (*Argas pusillus*) | Asiatic yellow house bats (*Scotophilus kuhlii* *temminckii*) | NR | S: KR537449.1  M: KR537448.1  L: KR537447.1 |
| soft tick bunyavirus (STBV) | Ticks (*Argas vespertilionis*) | Bats? | NR | S: LC027467.1  M: LC027466.1  L: LC027465.1 |
| Uzun-Agach virus (UZAV) | NK | Lesser mouse-eared myotis (*Myotis blythii oxygnathus*) | NR | TBD |

| **Putative species “*Kasokero nairovirus*”** |  |  |  |  |
| --- | --- | --- | --- | --- |
| Leopards Hill virus (LPHV) | NK | Giant leaf-nosed bats (*Hipposideros gigas*) | NR | S: NC_025833.1  M: NC_025832.1  L: NC_025831.1 |
| Kasokero virus (KAS[O]V) | NK | Egyptian rousettes (*Rousettus aegyptiacus*) | Yes (rare): mild to severe laboratory infections characterized by abdominal pain, diarrhea, fever, headache, and severe myalgia and arthralgia (nonlethal) | Coding-complete  S: KR537446.1  M: KR537445.1  L: KR537444.1 |
| Yogue virus (YOGV) | NK | Egyptian rousettes (*Rousettus aegyptiacus*) | NR | Coding-complete:  S: KR537455.1  M: KR537454.1  L: KR537453.1 |
| **Putative species “*Burana nairovirus*”** |  |  |  |  |
| Burana virus (BURV) | Ticks (*Haemaphysalis concinna*, *H. punctata*) | Domestic cows? | NR | Not yet deposited |
| Huángpí tick virus 1 (HTV-1) | Ticks (*Haemaphysalis doenitzi*) | NK | NR | S: KM817734.1  M: KM817706.1  L: KM817667.1 |
| Tǎchéng tick virus 1 (TTV-1) | Ticks (*Dermacentor marginatus*) | NK | NR | S: KM817743.1  M: KM817717.1  L: KM817683.1 |
| Tamdy virus (TDYV) | Ticks (*Haemaphysalis* *anatolicum*,  *H. asiaticum*, *H. concinna*, *Hyalomma marginatum*, *Rhipicephalus turanicus*) | Birds: white wagtails (*Motacilla alba*), European rollers (*Coracias garrulus*), hoopoes (*Upupa epops*), common starlings (*Sturnus vulgaris*), southern grey shrikes (*Lanius meriodinalis*)  Carnivores: steppe polecats (*Mustela eversmanni*)  Rodents: great gerbils (*Rhombomys opimus*)  Unspecified bats  Domestic sheep? | Yes (rare): arthralgia, fever, myalgia (nonlethal) | Not yet deposited |
| Wēnzhōu tick virus (WTV) | Ticks (*Haemaphysalis hystricis*) | NK | NR | S: KM817745.1  M: KM817718.1  L: KM817685.1 |
| **Putative ungrouped nairo-like viruses** |  |  |  |  |
| Artashat virus (ARTSV) | Ticks (*Ornithodoros alactagalis*, *O. verrucosus*) | Persian jirds (*Meriones persicus*), small five-toed jerboas (*Allactaga elater*)? | NR | TBD |
| Nàyǔn tick virus | Ticks (*Rhipicephalus* sp.) | NK | NR | TBD |
| Sānxiá water strider virus 1  (SWSV-1) | Water striders (gerrids) | NK |  | S: KM817737.1  M: KM817711.1  L: KM817674.1 |
| Shāyáng spider virus 1 | Spiders (*Neoscona nautica, Parasteatoda tepidariorum, Plexippus setipes*) | NK | NR | S: KM817738.1  M: KM817712.1  L: KM817676.1 |
| South Bay virus (SBV) | Ticks (*Ixodes scapularis*) | NK | NR | S: KM048321.1  L: KM048320.1 |
| Wǔhàn millipede virus 2 | Millipedes (polydesmids) | NK | NR | S: KM817757.1  L: KM817696.1 |
| Xīnzhōu spider virus (XSV) | Spiders (*Neoscona nautica, Parasteatoda tepidariorum*) | NK | NR | S: KM817762.1  M:KM817729.1 (incomplete)  L: KM817702.1 |

**Table S2. M-segment polyprotein descriptions. Each species column depicts averages of its members.** Sample sizes are: Crimean-Congo hemorrhagic fever
nairovirus n = 1, “Hazara nairovirus” n = 2, Dugbe nairovirus n = 5, Sakhalin nairovirus n = 4, Thiafora nairovirus n = 2, “Kasokero nairovirus” n = 3, Qalyub nairovirus
n = 2, “Keterah nairovirus” n = 2, Dera Ghazi Khan nairovirus n = 5, Hughes nairovirus n = 7, and “Burana nairovirus” n = 3. Averages for predicted characteristics were rounded up. Proposed new taxa are highlighted in red and placed in quotation marks.

| Predicted Glycoprotein Precursor Processing | *Crimean-Congo hemorrhagic fever nairovirus* | *“Hazara nairovirus*” | *Dugbe nairovirus* | *Sakhalin nairovirus* | | *Thiafora nairovirus* | | “*Kasokero nairovirus*” | | *Qalyub nairovirus* | “*Keterah nairovirus*” | *Dera Ghazi Khan nairovirus* | *Hughes nairovirus* | “*Burana nairovirus*” |
| --- | --- | --- | --- | --- | --- | --- | --- | --- | --- | --- | --- | --- | --- | --- |
| Polyprotein Mass (deduced kDa) | 187 | 158 | 173 | | 158 | | 144 | | 159 | 164 | 183 | 157 | 155 | 150^φ^ |
| Glycoproteins^¥^ | 5 | 3 | 3 | | 3 | | 3 | | 2 | 4 | 4 | 3 | 2 | 3 |
| Conserved Cleavage Sites | RSKR, RKPL, RRLL, RKLL | RRLL, RKLL | RKPL, RRLL, RKLL | | RRLL, RKLL, RKPL | | RRLL, RKLL | | RRLL, RKLL | RKPL, RKLL | RKLL | RRLL, RKLL | RRLL, RKLL | RRLL, RKLL, RKPL |
| *O*-Linked Glycosylations | 100 | 29 | 38 | | 35 | | 33 | | 38 | 48 | 135 | 17 | 4 | 38 |
| *N*-Linked Glycosylations | 11 | 6 | 7 | | 17 | | 12 | | 12 | 12 | 20 | 12 | 12 | 9 |
| Transmembrane domains (TMDs) | 5 | 5 | 5 | | 3 | | 3 | | 4 | 2^¤¤¤^ | 3 | 3 | 4 | 3 |
| Glycosylated Regions/Mucin-like domains (MLDs) | 1 | 1 | 1 | | 1 | | 1 | | 1 | 1 | 2–3 | 1 | None | 1 |
| MLD Mass (deduced kDa) | 23 | 3–5 | 11 | | 3–10 | | 7 | | 8–11 | 10–12 | 8–37 | 3–5 | 0 | 8–11 |

^¤¤¤^A single member of this species group (*Qalyub nairovirus*) is predicted to contain only a single transmembrane region. ^¥^ No differentiation of structural versus non-structural glycoproteins. ^φ^ Two members of the species *Burana nairovirus* are predicted to have two open reading frames on the M-segment encoding a separate stand-alone glycoprotein next to the polyprotein (GPC).

References

1. Plyusnin, A.; Beaty, B. J.; Elliott, R. M.; Goldbach, R.; Kormelink, R.; Lundkvist, A.; Schmaljohn, C. S.;
   Tesh, R. B., Family *Bunyaviridae*. In *Virus Taxonomy - Ninth Report of the International Committee on Taxonomy of Viruses*, King, A. M. Q.; Adams, M. J.; Carstens, E. B.; Lefkowitz, E. J., Eds. Elsevier/Academic Press: London, United Kingdom, 2011; pp 725‒741.
2. Львов, Д. К.; Альховский, С. В.; Щелканов, М. Ю.; Щетинин, А. М.; Аристова, В. А.; Морозова, Т. Н.; Гительман, А. К.; Дерябин, П. Г.; Ботиков, А. Г., [L'vov, D. K.; Al'hovskij, S. V.; Shhelkanov, M. Ju.; Shhetinin, A. M.; Aristova, V. A.; Morozova, T. N.; Gitel'man, A. K.; Derjabin, P. G.; Botikov, A. G.], Таксономия ранее не классифицированного вируса ЧИМ (CHIMV - Chim virus) (*Bunyaviridae*, *Nairovirus*, группа Кальюб), изолированного в Узбекистане и Казахстане из иксодовых (*Acari*: *Ixodidae*) и аргасовых (*Acari*: *Argasidae*) клещей, собранных в норах больших песчанок *Rhombomys opimus* Lichtenstein, 1823 (*Muridae*, *Gerbillinae*) [Taxonomic status of Chim virus (CHIMV) (*Bunyaviridae*, *Nairovirus*, Qalyub group) isolated from *Ixodidae* and *Argasidae* ticks collected from great gerbil (*Rhombomys opimus* Lichtenstein, 1823) (*Muridae*, *Gerbillinae*) burrows in Uzbekistan and Kazakhstan]. *Vopr. Virusol.* **2014**, *59*, 18‒23 [Russian].
3. Crabtree, M. B.; Sang, R.; Miller, B. R., Kupe virus, a new virus in the family *Bunyaviridae*, genus *Nairovirus*, kenya. *Emerg. Infect. Dis.* **2009**, *15*, 147‒54.
4. Lvov, D. K.; Shchelkanov, M. Y.; Alkhovsky, S. V.; Deryabin, P. G., Single-stranded RNA viruses. In *Zoonotic Viruses of Northern Eurasia: Taxonomy and Ecology. Taxonomy and Ecology*, Elsevier/Academic Press: Amsterdam, The Netherlands, 2015; pp 135‒392.
5. 5. Альховский, С. В.; Львов, Д. К.; Щелканов, М. Ю.; Щетинин, А. М.; Дерябин, П. Г.; Самохвалов, Е. И.; Гительман, А. К.; Ботиков, А. Г., [Al'hovskij, S. V.; L'vov, D. K.; Ŝelkanov, M. Û.; Ŝetinin, A. M.; Derâbin, P. G.; Samohvalov, E. I.; Gitel'man, A. K.; Botikov, A. G.], Таксономия вируса Хасан (Khasan, KHAV) – нового вируса рода *Phlebovirus* (сем. *Bunyaviridae*), изолированного из клещей *Haemaphysalis longicornis* (Neumann, 1901) в Приморском крае (Россия) [The taxonomy of the Khasan virus (KHAV), a new representative of the *Phlebovirus* genus (*Bunyaviridae*), isolated from *Haemaphysalis longicornis* (Neumann, 1901) ticks in the Maritime Territory (Russia)]. *Vopr. Virusol.* **2013**, *58,* 15‒8.
6. Sonenshine, D. E.; Roe, M. R., *Biology of ticks*. 2nd ed.; Oxford University Press: 2013; Vol. 2.
7. Nuttall, P. A.; Carey, D.; Moss, S. R.; Green, B. M.; Spence, R. P., Hughes group viruses (*Bunyaviridae*) fro the seabird tick *Ixodes* (*Ceratixodes*) *uriae* (Acari, Ixodidae). *J Med Entomol* **1986**, *23*, 437‒440.
8. Moss, S. R.; Petersen, Æ.; Nuttall, P. A., Tick-borne viruses in Icelandic seabird colonies. *Acta Naturalia Islandica* **1986**, *32*, 1‒19.
9. Львов, Д. К.; Альховский, С. В.; Щелканов, М. Ю.; Щетинин, А. М.; Дерябин, П. Г.; Самохвалов, Е. И.; Гительман, А. К.; Ботиков, А. Г., [L'vov, D. K.; Al'hovskij, S. V.; Shhelkanov, M. Ju.; Shhetinin, A. M.; Derjabin, P. G.; Samohvalov, E. I.; Gitel'man, A. K.; Botikov, A. G.], Генетическая характеристика вируса Каспий (CASV - *Caspiy virus*) (*Bunyaviridae*, *Nairovirus*), изолированного от чайковых (Laridae Vigors, 1825) и крачковых (*Sternidae* Bonaparte, 1838) птиц и аргасовых клещей *Ornithodoros capensis* Neumann, 1901 (*Argasidae* Koch, 1844) на западном и восточном побережьях Каспийского моря [Genetic characterization of *Caspiy virus* (CASV) (*Bunyaviridae*, *Nairovirus*) isolated from Laridae (Vigors, 1825) and *Sternidae* (Bonaparte, 1838) birds and *Argasidae* (Koch, 1844) *Ornithodoros capensis* Neumann, 1901, ticks form western and eastern coasts of the Caspian Sea]. *Vopr. Virusol.* **2014**, *59*, 24‒9 [Russian].
10. Dacheux, L.; Cervantes-Gonzalez, M.; Guigon, G.; Thiberge, J.-M.; Vandenbogaert, M.; Maufrais, C.; Caro, V.; Bourhy, H., A preliminary study of viral metagenomics of French bat species in contact with humans: identification of new mammalian viruses. *PLoS One* **2014**, *9*, e87194.
11. Альховский, С. В.; Львов, Д. К.; Щелканов, М. Ю.; Щетинин, А. М.; Дерябин, П. Г.; Гительман, А. К.; Ботиков, А. Г.; Самохвалов, Е. И.; Закарян, В. А., [Al'hovskij, S. V.; L'vov, D. K.; Ŝelkanov, M. Û.; Ŝetinin, A. M.; Derâbin, P. G.; Gitel'man, A. K.; Botikov, A. G.; Samohvalov, E. I.; Zakarân, V. A.], Таксономия вируса Арташат (ARTSV -- Artashat virus) (*Bunyaviridae*, *Nairovirus*), изолированного из клещей *Ornithodoros alactagalis* Issaakjan, 1936 и *O. verrucosus* Olenev, Sassuchin et Fenuk, 1934 (*Argasidae* Koch, 1844), собранных в Закавказье [Taxonomic status of Artashat virus (ARTSV) (*Bunyaviridae*, *Nairovirus*) isolated from *Ornithodoros alactagalis* Issaakjan, 1936 and *O. verrucosus* Olenev, Sassuchin et Fenuk, 1934 ticks (*Argasidae* Koch, 1844) collected in Transcaucasia]. *Vopr. Virusol.* **2014**, *59*, 24‒8 [Russian].
12. Альховский, С. В.; Львов, Д. К.; Щелканов, М. Ю.; Щетинин, А. М.; Дерябин, П. Г.; Самохвалов, Е. И.; Гительман, А. К.; Ботиков, А. Г., [Al'hovskij, S. V.; L'vov, D. K.; Ŝelkanov, M. Û.; Ŝetinin, A. M.; Derâbin, P. G.; Samohvalov, E. I.; Gitel'man, A. K.; Botikov, A. G.], Таксономия вируса Иссык-Куль (Issyk-kul virus, ISKV; *Bunyaviridae*, *Nairovirus*), возбудителя Иссык-Кульской лихорадки, изолированного от летучих мышей (*Vespertilionidae*) и клещей *Argas* (*Carios*) *vespertilionis* (Latreille, 1796) [Taxonomy of Issyk-kul virus (ISKV, *Bunyaviridae*, *Nairovirus*), the etiologic agent of Issyk-kul fever isolated from bats (*Vespertilionidae*) and *Argas* (*Carios*) *vespertilionis* (Latreille, 1796) ticks]. *Vopr. Virusol.* **2013**, *58*, 11‒5 [Russian].
13. Atkinson, B.; Marston, D. A.; Ellis, R. J.; Fooks, A. R.; Hewson, R., Complete genomic sequence of Issyk-kul virus. *Genome Announc.* **2015**, *3*, e00662-15.
14. Walker, P. J.; Widen, S. G.; Firth, C.; Blasdell, K. R.; Wood, T. G.; Travassos da Rosa, A. P. A.; Guzman, H.; Tesh, R. B.; Vasilakis, N., Genomic characterization of Yogue, Kasokero, Issyk-Kul, Keterah, Gossas, and Thiafora viruses: nairoviruses naturally infecting bats, shrews, and ticks. *Am. J. Trop. Med. Hyg.* **2015**, *93*, 1041‒51.
15. Oba, M.; Omatsu, T.; Takano, A.; Fujita, H.; Sato, K.; Nakamoto, A.; Takahashi, M.; Takada, N.; Kawabata, H.; Ando, S.; Mizutani, T., A novel Bunyavirus from the soft tick, *Argas vespertilionis*, in Japan. *J Vet. Med. Sci.* **2016**, *78*, 443‒5.
16. Ishii, A.; Ueno, K.; Orba, Y.; Sasaki, M.; Moonga, L.; Hang'ombe, B. M.; Mweene, A. S.; Umemura, T.; Ito, K.; Hall, W. W.; Sawa, H., A nairovirus isolated from African bats causes haemorrhagic gastroenteritis and severe hepatic disease in mice. *Nat. Commun.* **2014**, *5*, 5651.
17. Львов, Д. К.; Альховский, С. В.; Щелканов, М. Ю.; Щетинин, А. М.; Аристова, В. А.; Гительман, А. К.; Дерябин, П. Г.; Ботиков, А. Г., [Львов, Д. К.; Альховский, С. В.; Щелканов, М. Ю.; Щетинин, А. М.; Аристова, В. А.; Гительман, А. К.; Дерябин, П. Г.; Ботиков, А. Г.], Таксономия ранее негруппированного вируса Тамды (TAMV-Tamdy virus) (*Bunyaviridae*, *Nairovirus*), изолированного от иксодовых клещей *Hyalomma asiaticum asiaticum* Schülce et Schlottke, 1929 (*Ixodidae*, *Hyalomminae*) в Средней Азии и Закавказье [Taxonomy of previously unclassified Tamdy virus (TAMV) (*Bunyaviridae*, *Nairovirus*) isolated from *Hyalomma asiaticum asiaticum* Schülce et Schlottke, 1929 (*Ixodidae*, *Hyalomminae*) ticks in the Middle East and Transcaucasia]. *Vopr. Virusol.* **2014**, *59*, 15‒22 [Russian].
18. Львов, Д. К.; Альховский, С. В.; Щелканов, М. Ю.; Щетинин, А. М.; Дерябин, П. Г.; Гительман, А. К.; Аристова, В. А.; Ботиков, А. Г., [L'vov, D. K.; Al'hovskij, S. V.; Shhelkanov, M. Ju.; Shhetinin, A. M.; Derjabin, P. G.; Gitel'man, A. K.; Aristova, V. A.; Botikov, A. G.], Таксономический статус вируса Бурана (BURV - Burana virus) (*Bunyaviridae*, *Nairovirus*, *группа Тамды*), изолированного из клещей *Haemaphysalis punctata* Canestrini et Fanzago, 1877 и *Haem. concinna* Koch, 1844 (*Ixodidae*, *Haemaphysalinae*) в Кыргызстане [Taxonomic status of Burana virus (BURV) (*Bunyaviridae*, *Nairovirus*, Tamdy group) isolated from *Haemaphysalis punctata* Canestrini et Fanzago, 1877 and *Haem. concinna* Koch, 1844 ticks (*Ixodidae*, *Haemaphysalinae*) in Kyrgyzstan]. *Vopr. Virusol.* **2014**, *59*, 10‒5 [Russian].
19. Li, C. X.; Shi, M.; Tian, J. H.; Lin, X. D.; Kang, Y. J.; Chen, L. J.; Qin, X. C.; Xu, J.; Holmes, E. C.; Zhang, Y. Z., Unprecedented genomic diversity of RNA viruses in arthropods reveals the ancestry of negative-sense RNA viruses. *Elife* **2015**, *4*, e05378.
20. Xia, H.; Hu, C.; Zhang, D.; Tang, S.; Zhang, Z.; Kou, Z.; Fan, Z.; Bente, D.; Zeng, C.; Li, T., Metagenomic profile of the viral communities in *Rhipicephalus* spp. ticks from Yunnan, China. *PLoS One* **2015**, *10*, e0121609.
21. Tokarz, R.; Williams, S. H.; Sameroff, S.; Sanchez Leon, M.; Jain, K.; Lipkin, W. I., Virome analysis of *Amblyomma americanum*, *Dermacentor variabilis*, and *Ixodes scapularis* ticks reveals novel highly divergent vertebrate and invertebrate viruses. *J. Virol.* **2014**, *88*, 11480‒92.
22. Альховский, С. В.; Львов, Д. К.; Щелканов, М. Ю.; Дерябин, П. Г.; Щетинин, А. М.; Самохвалов, Е. И.; Аристова, В. А.; Гительман, А. К.; Ботиков, А. Г., [Al'hovskij, S. V.; L'vov, D. K.; Ŝelkanov, M. Û.; Derâbin, P. G.; Ŝetinin, A. M.; Samohvalov, E. I.; Aristova, V. A.; Gitel'man, A. K.; Botikov, A. G.], Генетическая характеристика вируса Узун-Агач (UZAV - Uzun-Agach virus) (*Bunyaviridae*, *Nairovirus*), изолированного в Казахстане от остроухой ночницы *Myotis blythii oxygnathus* Monticelli, 1885 (*Chiroptera*; *Vespertilionidae*) [Genetic characterization of Uzun-Agach virus (UZAV, *Bunyaviridae*, *Nairovirus*), isolated from *Myotis blythii* *oxygnathus* Monticelli, 1885 bats (*Chiroptera*; *Vespertilionidae*) in Kazakhstan]. *Vopr. Virusol.* **2014**, *59*, 23‒6 [Russian].
23. Walker, P. J.; Widen, S. G.; Wood, T. G.; Guzman, H.; Tesh, R. B.; Vasilakis, N., A global genomic characterization of nairoviruses identifies nine discrete genogroups with distinctive structural characteristics and host-vector associations. *Am. J. Trop. Med. Hyg.* **2016**, *94*, 1107‒22.
